# Changes in Kitchen Thermal Exposure Following the Introduction of Electric Cooking: A Before-and-After Case Study of Three Kampala Kitchens

**DOI:** 10.3390/ijerph23081034

**Published:** 2026-08-07

**Authors:** Rihab Khalid, Agnes Naluwagga, Jimmy Agaba, Adrian Okorio, Charles Kayemba

**Affiliations:** 1 MECS Programme, Geography and Environment Department, Loughborough University, Loughborough LE11 3TU, UK; 2 Centre for Research in Energy and Energy Conservation (CREEC), Plot 7 Cornwall Crescent, K10 Village, Kyambogo Lower Estate, Nakawa Division, Kampala P.O. Box 7062, Uganda; analuwagga@creec.or.ug (A.N.); jagaba@creec.or.ug (J.A.); aokorio@creec.or.ug (A.O.); ckayemba@creec.or.ug (C.K.)

**Keywords:** electric cooking, kitchen thermal exposure, Heat Index, occupational heat, institutional kitchens, before-and-after case study, Uganda

## Abstract

**Highlights:**

**Public health relevance—how does this work relate to a public health issue?**

- Institutional and commercial kitchens can function as indoor occupational heat risk environments, particularly where combustion-based cooking, high meal volumes and limited ventilation overlap.
- Cooking-related heat exposure is spatially uneven, with the highest apparent heat burden concentrated near the cookstove, alongside smaller but meaningful impacts across the ambient kitchen environment.

**Public health significance—why is this work of significance to public health?**

- Across three Kampala kitchens, documented active e-cooking days were associated with lower temperatures, lower apparent heat burden and less time in higher Heat Index risk categories.
- The study links environmental monitoring with cooking diaries, repeated thermal comfort and sensation votes and observations, allowing environmental measurements to be interpreted alongside workers’ reported experience and coping practices.

**Public health implications—what are the key implications or messages for practitioners, policy makers and/or researchers in public health?**

- Clean cooking interventions should be assessed for their effects on heat exposure, thermal comfort and occupational wellbeing, alongside emissions, energy use and cost.
- E-cooking may contribute to safer and healthier kitchen workplaces when supported by reliable electricity, safe wiring, suitable appliances, ventilation and appropriate kitchen design; the present study does not measure physiological heat strain.

**Abstract:**

Institutional and commercial cooks may experience sustained heat exposure from combustion-based cooking, yet field evidence on thermal conditions following the introduction of electric cooking remains limited. This exploratory before-and-after case study examined three purposively selected kitchens in Kampala, Uganda, combining five-minute temperature and relative humidity monitoring at three spatial positions with cooking diaries, repeated thermal comfort and sensation votes, and enumerator observations. Heat Index (HI) was used as a temperature–humidity screening proxy, and Kampala weather data were used to calculate weather-adjusted differences. On documented active e-cooking days, mean hourly near-stove HI fell from 33.26 °C to 26.00 °C at Site A, 31.35 °C to 29.55 °C at Site B, and 31.71 °C to 27.50 °C at Site C. Time in the Safe HI category increased from 23.5% to 67.8%, 17.0% to 33.3%, and 25.4% to 53.0%, respectively. Day-level tests and a sensitivity analysis using all weather-matched intervention period logger days showed the same overall direction. Raw cooking durations declined for several meals, although, after normalisation by the number of people served, the reductions remained statistically detectable only for Site B breakfast and lunch. The results provide exploratory evidence of lower environmental thermal exposure under documented active e-cooking conditions. Sequential timing, purposive sampling, workload differences, incomplete intervention records and the absence of radiant, air speed and physiological measurements prevent causal interpretation as reduced occupational heat strain.

## 1. Introduction

Heat stress is increasingly recognised as a major urban and occupational public health risk, intensified by climate change, rapid urbanisation, and the urban heat island effect [1,2,3]. Heat stress refers to physiological strain that arises when total heat load exceeds the body’s capacity to dissipate heat, with potential consequences for health, safety and work performance [2]. In this paper, environmental thermal exposure concerns the physical conditions surrounding the worker; thermal comfort and sensation describe subjective experience; and physiological heat strain concerns bodily responses such as elevated core temperature, cardiovascular load, sweating and dehydration. Studies of cooks have documented such physiological responses [4], but environmental and self-report measures cannot be treated as direct measurements of physiological strain. Recent global guidance from the World Health Organization and World Meteorological Organization identifies workplace heat stress as a growing climate-related challenge [2]. Evidence suggests that labour productivity declines as occupational heat exposure increases [2]. The International Labour Organization likewise frames heat at work as a major occupational safety and health concern requiring workplace-level measures, policy attention and employer responsibility [3]. Recent public health reviews associate occupational heat exposure with heat-related illness, injury risk, fatigue, reduced performance and mortality [5].

Within this broader landscape, kitchens represent a particularly intense heat exposure micro-environment [6]. Heat exposure here refers to the thermal conditions to which workers are subjected during cooking, and heat burden refers to the magnitude or apparent intensity of that exposure in the kitchen environment, as captured by heat metrics. Cooking produces substantial sensible and radiant heat, often alongside elevated humidity, and these loads are superimposed on already warm ambient conditions. In many low- and middle-income settings, kitchen heat exposure is further shaped by limited mechanical ventilation, compact layouts, high occupancy during peak service, and continued reliance on combustion-based fuels and appliances [7,8,9]. Cooking and health research has historically focused on household air pollution and emissions exposure [10,11,12]. However, recent public health guidance on the combined risks of heat stress and air pollution reinforces the need to examine how thermal and pollutant exposures may intersect in everyday environments such as kitchens [13]. Cooking-related heat exposure and thermal discomfort therefore remain under-addressed in clean cooking, occupational health and urban heat resilience debates [14].

This gap is especially important in institutional and commercial kitchens. These spaces often operate outside the assumptions embedded in conventional thermal comfort standards and models. Commercial kitchens are characterised by concentrated heat sources, strong radiant loads, moisture generation, variable ventilation, and marked spatial and temporal heterogeneity. Under such conditions, room-scale indices such as Predicted Mean Vote (PMV) and Predicted Percentage of Dissatisfied (PPD) may not adequately represent conditions near cooking lines, hoods or other localised hotspots [6,15,16]. Existing evidence indicates that cooking technology influences measured thermal conditions and workers’ reported thermal experience [15,16].

In African contexts, where polluting fuels remain widespread [17], recent occupational health evidence suggests that more than two-thirds of hospitality kitchen workers report heat-stress-related symptoms, with wood fuel use, poor ventilation, and higher Heat Index values among the associated factors [18]. Hence, kitchen heat exposure becomes not just a matter of discomfort, but an occupational health concern shaped by fuel choice, infrastructure, and workplace conditions. Yet field-based evidence from sub-Saharan Africa remains limited, particularly for urban institutional and commercial kitchens where cooking volumes, exposure durations, and operational demands can be high.

Uganda provides a particularly relevant setting in which these dynamics converge. Cooking remains predominantly biomass-based: around 68% of households use firewood, most of the remainder use charcoal, and LPG and electricity still play only minor roles nationally (at under 2%) [19]. In Kampala, charcoal use is especially dominant (reported at 82% of households), reflecting the urbanisation of biomass dependence rather than its displacement [19]. Institutional cooking likewise remains overwhelmingly biomass-based, with 49% of institutions using charcoal as their main cooking fuel, 21% using firewood, and only 2.5% using electricity [19]. At the same time, electricity access and reliability constraints continue to shape the feasibility of transitions to electric cooking (e-cooking). These conditions make Kampala an important context for examining how cooking energy transitions may alter the kitchen thermal environment (i.e., the measured temperature, humidity, and their spatial distribution within the kitchen) and occupational heat exposure.

Against this background, this paper addresses two linked gaps. First, field-based evidence remains limited on how thermal conditions change when electric appliances are introduced into working institutional and commercial kitchens in sub-Saharan Africa. Second, few studies combine spatially resolved monitoring with fuel and appliance use, workflow, repeated worker reports and implementation constraints. Occupational heat research increasingly emphasises the need to connect environmental exposure with worker experience and organisational conditions [5,20]. Mixed-methods research is valuable in this setting because it can relate high-frequency thermal measurements to work routines and reported experience, while revealing possible observer, translation and desirability influences on subjective responses [21].

This exploratory case study therefore examines how temperature, relative humidity and apparent heat burden differed between a combustion-based baseline period and documented active e-cooking days in three Kampala kitchens; how differences in meal volume and cooking duration affected interpretation; and how cooks described their thermal experience under the two observed conditions. The study evaluates changes in environmental thermal exposure and subjective experience. It does not test the physiological efficacy of e-cooking as a heat strain intervention.

The study addresses four questions:

1. How did near-stove, indoor ambient and near-kitchen outdoor temperature, relative humidity and Heat Index differ between the baseline period and documented active e-cooking days at each site?
2. How did cooking duration and the number of people served differ between phases, and did differences in cooking time persist after normalisation by the number of people served?
3. How did cooks’ repeated thermal sensation and comfort reports differ between baseline and documented active e-cooking meals, and what implementation factors shaped interpretation of these reports?
4. Were the weather-adjusted thermal findings directionally similar when all weather-matched intervention period logger days were included?

Section 2 reviews research on kitchen thermal environments and cooking-related heat exposure. Section 3 describes the study sites and methods. Section 4 presents the findings. Section 5 concludes with implications for research and practice.

## 2. Cooking and Thermal Comfort in Kitchens

Kitchens, particularly institutional and commercial kitchens, sit uneasily within both mainstream thermal comfort research and conventional occupational heat stress guidance. Thermal comfort frameworks such as ASHRAE 55 [22] and ISO 7730 [23] conceptualise comfort as a subjective response to the interaction of air temperature, radiant temperature, humidity, air speed, clothing and activity, whereas occupational health guidance treats heat as a hazard when metabolic and environmental heat loads exceed the body’s ability to dissipate heat [2,24]. Recent public health research also emphasises the value of heat stress indices for occupational risk assessment, while recognising that index-based measures remain partial representations of workers’ exposure, vulnerability and work organisation [5,25]. These perspectives are complementary, but neither fully captures the environmental complexity of kitchen work, where workers are exposed to concentrated heat sources, transient peaks, uneven thermal fields and task-specific radiant loads.

Kitchens often combine high activity levels (metabolic heat), strong localised radiant loads, moisture generation from cooking processes, such as boiling, frying and simmering, and dynamic airflow created by extraction hoods, make-up air and openings [26,27]. As a result, average room comfort metrics do not account for hotspots and spatial heterogeneity of kitchen workflows. Experimental, modelling and field studies show that kitchens often function as thermal microclimates, with marked spatial and temporal variation in temperature, humidity and radiant asymmetry [26,27,28]. Recent work on Chinese-style residential kitchens further shows that cooks’ thermal comfort is shaped by the interaction between the cooking method, thermal environment and behavioural patterns, reinforcing the need for task-sensitive assessment of kitchen heat exposure [29]. These findings are especially relevant for institutional and commercial kitchens, where exposure is often prolonged, repetitive and concentrated around specific cooking stations.

A key implication is that conventional comfort models may be poorly suited to kitchen environments. Simone et al.’s [6] large study of more than 100 commercial kitchens showed that workers were frequently exposed to warm-to-hot conditions outside the normal scope of ASHRAE 55 and ISO 7730, and that PMV/PPD was not directly applicable across the observed conditions. Related work in school kitchens similarly found that a substantial share of sites exceeded recommended thermal limits for moderate and heavy work [30]. Building environment studies point in the same direction: cooking can raise kitchen air temperatures by around 4.0–11.5 K depending on dish type and operation, and even with an exhaust hood, pollutant concentrations and thermal loads can remain high in the breathing zone [31]. Earlier studies on kitchen appliance plumes also highlighted the importance of appliance-specific heat release and airflow behaviour for kitchen environmental performance [32].

Evidence from low- and middle-income settings is growing but remains uneven across regions and kitchen types. In Ethiopia, a large cross-sectional study of hospitality kitchen workers reported a high prevalence of heat-stress-related symptoms and found associations with workload, poor ventilation, wood fuel use, and higher Heat Index values [18]. Recent evidence from school food service workers in Korea similarly reports substantial daily heat exposure and a high prevalence of heat-related illness symptoms, with longer exposure duration associated with greater symptom risk [33]. Studies from Nigeria [34] and multi-city low-income household settings [35] similarly report elevated temperature and humidity during cooking, with many kitchens exceeding conventional comfort thresholds and a substantial proportion failing to meet ideal ventilation conditions. Indian studies of rural household kitchens and canteen kitchens similarly document high discomfort, inadequate ventilation and widespread dissatisfaction with prevailing thermal conditions during cooking periods [9,36,37]. Together, these studies show that kitchen heat stress is not confined to extreme climates or industrial settings but is a recurrent feature of everyday cooking environments across domestic, institutional and commercial settings.

At the same time, most clean cooking research has prioritised emission reduction and adoption rather than thermal exposure. Cooking technologies shape indoor thermal environments through their efficiency, heat gain to space, radiant exchange, moisture release and cooking duration [14,38,39]. Ventilation design, make-up air and appliance choice can materially influence pollutant removal and thermal comfort [26,27,39,40]. Comparative evidence also suggests that cooking fuel and appliance choice matter for thermal conditions and reported experience. Haruyama et al. [15] found greater subjective thermal strain in gas kitchens than in electric kitchens, consistent with higher ambient temperature, mean radiant temperature and WBGT. More recent work comparing electric and gas cooking in air-conditioned kitchens reports more favourable thermal conditions under electric cooking [16,41]. However, much of this evidence is cross-sectional, laboratory-based or focused on residential kitchens, leaving limited intervention-based evidence from working institutional and commercial kitchens.

A final limitation in the literature is methodological. Kitchen studies often rely on either environmental monitoring and modelling or worker surveys, with fewer studies integrating measured conditions with lived experience in a time-sensitive way [15,36]. Subjective scales show that workers’ perceptions do not always map neatly onto standardised comfort formulations under transient and spatially uneven conditions [28,42]. Occupational heat research also emphasises workers’ perceptions, workplace routines and organisational barriers to prevention [5,20]. Mixed-method designs combining high-frequency monitoring with diaries, repeated perception prompts and structured observation can relate thermal experience to tasks, locations and cooking technologies [43]. These self-reports must still be interpreted cautiously because observation, training, novelty and perceived project expectations may influence responses.

Against this background, two gaps remain especially important. First, there is very limited intervention-based evidence on changes in thermal exposure following a shift in cooking technology in real institutional and commercial kitchens in sub-Saharan Africa. Second, there is limited research combining spatially sensitive environmental monitoring with worker experience, workload and operational constraints. The present study addresses these gaps as an exploratory case study of environmental thermal exposure; it does not estimate a population-wide or physiological treatment effect.

## 3. Materials and Methods

### 3.1. Study Design and Sites

This study used an exploratory, quasi-experimental before-and-after case study design to examine changes in kitchen thermal exposure following the introduction of electric cooking appliances in three institutional and commercial kitchens in Kampala, Uganda. Each kitchen provided its own temporal comparator. The three kitchens were purposively selected after pre-visits to 11 potential sites. Operational criteria included predominant use of combustion-based fuels at baseline, meal volume and diversity, electrical infrastructure feasibility, access for repeated monitoring, and willingness of management and staff to participate. The resulting sample was intended to provide contrasting implementation cases rather than a representative sample of Kampala kitchens. Table 1 summarises the three study sites.

The design did not include a parallel control kitchen, randomised intervention timing or crossover. Consequently, phase differences may reflect weather, building thermal response, workload, ventilation behaviour, workflow or other unmeasured temporal factors as well as the change in cooking technology. The analyses are therefore site-specific and exploratory rather than estimates of a causal treatment effect.

**Figure 1:**
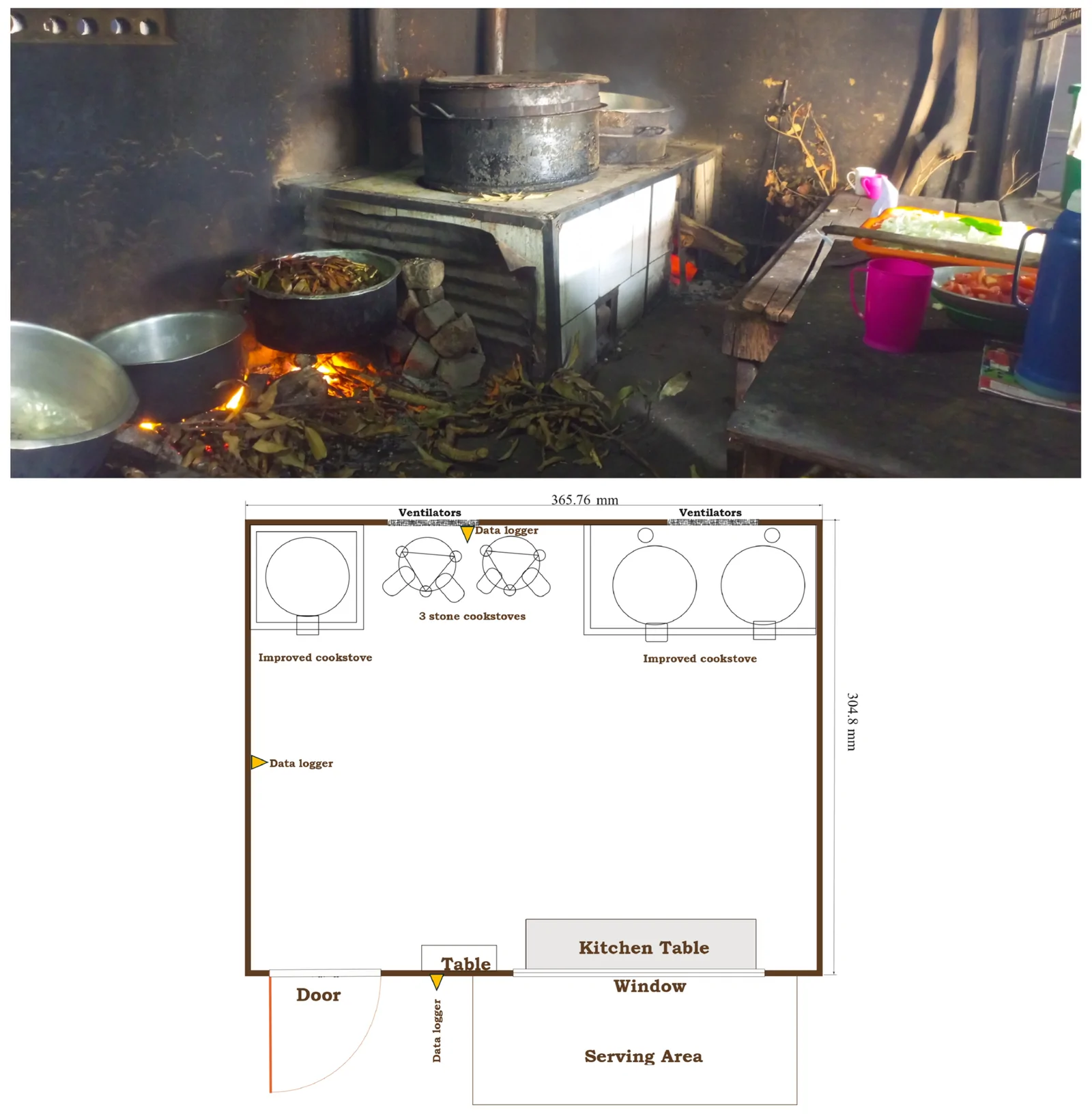
Site A—High school kitchen layout and interior view.

Site A (Figure 1) was an institutional high school kitchen serving approximately 50–100 staff and students, located in a built-up inner-suburban area of Kampala, relying mainly on firewood, using traditional three-stone stoves alongside some improved cookstoves.

**Figure 2:**
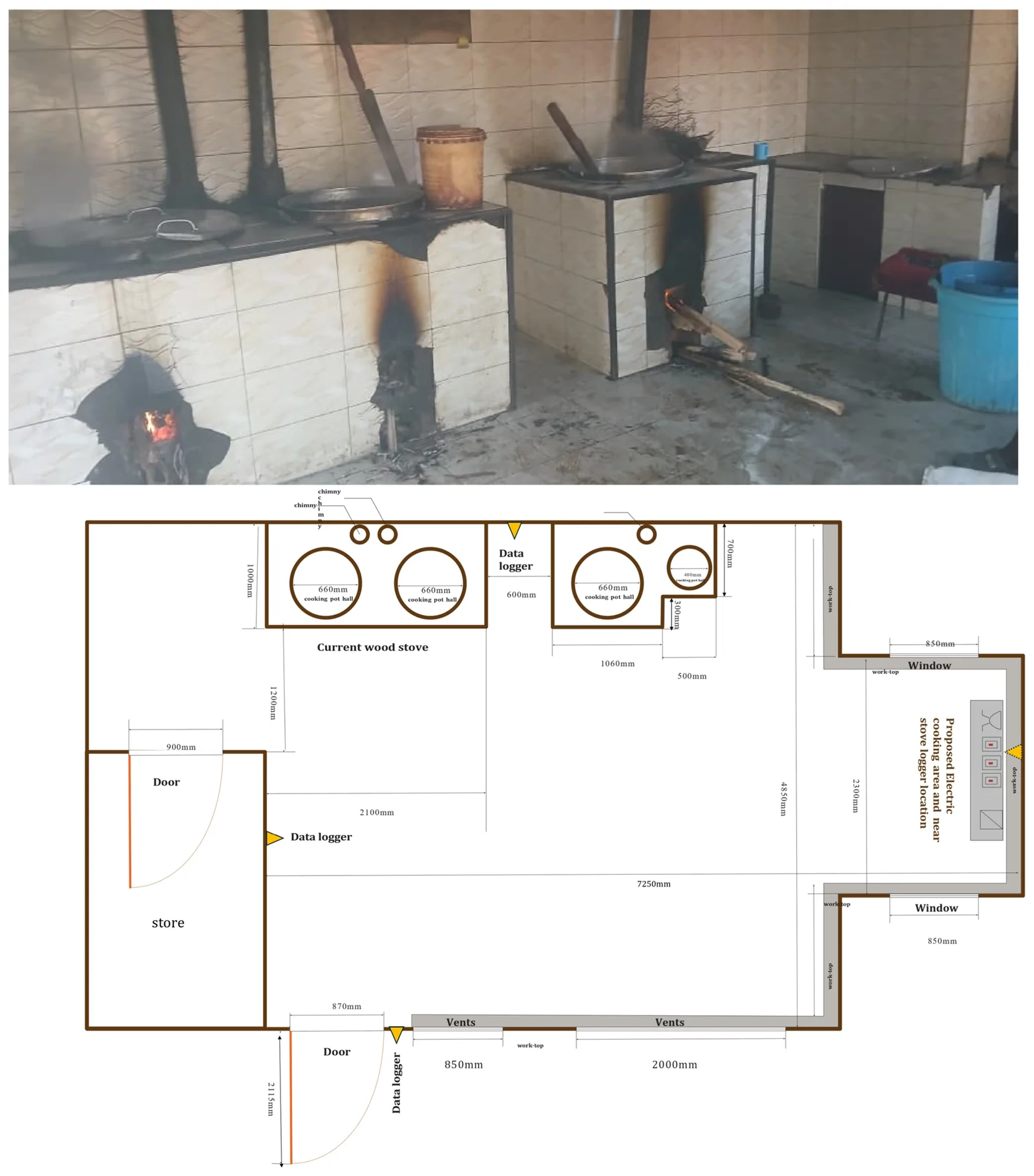
Site B—remand home kitchen layout and interior view.

Site B (Figure 2) was a remand home kitchen preparing meals for approximately 50–250 residents, situated approximately 5 km from the city centre in a densely developed urban setting. One or two staff typically prepare three meals per day using improved cookstoves, with firewood as the primary fuel.

**Figure 3:**
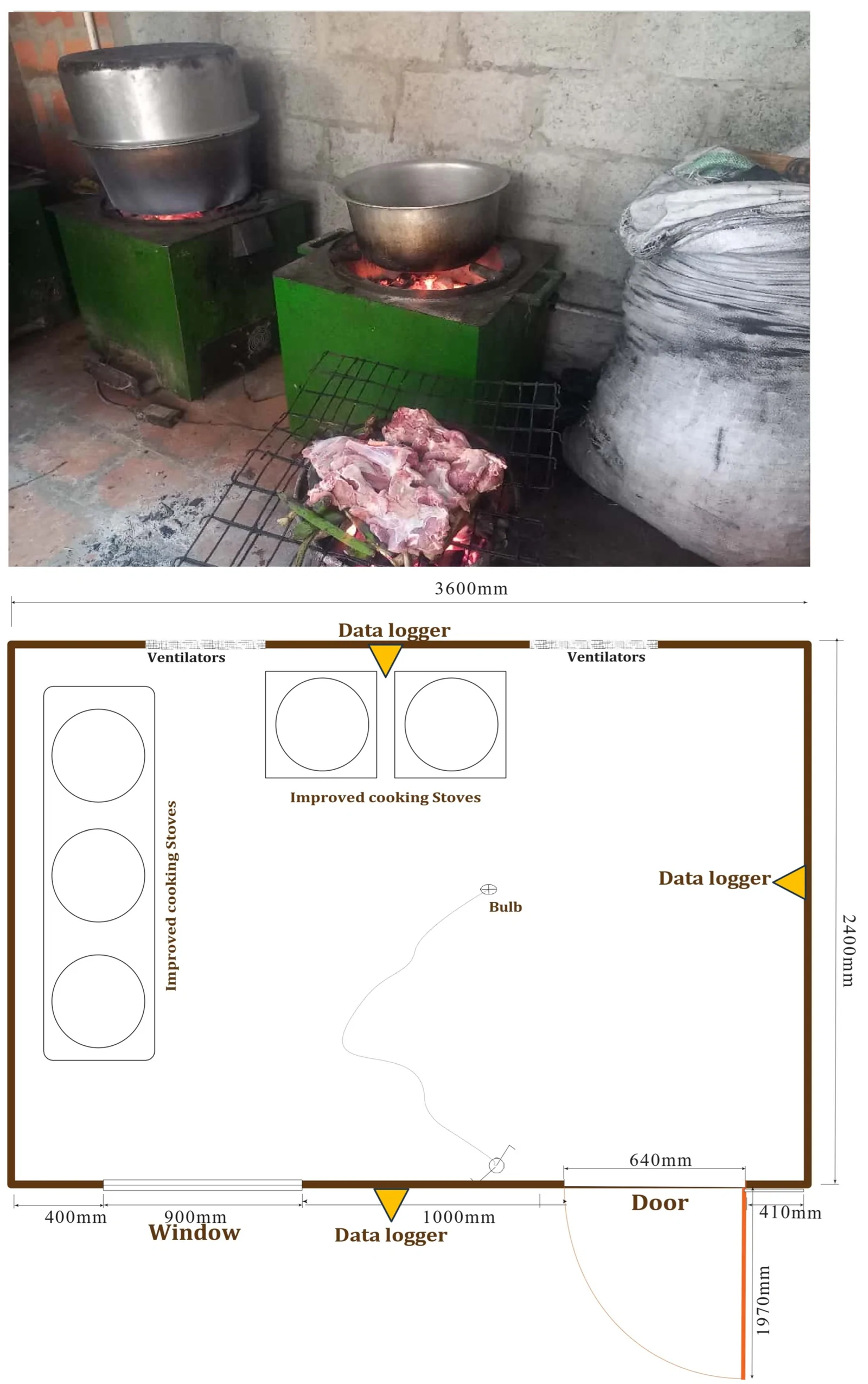
Site C—commercial kitchen layout and interior view.

Site C (Figure 3) was a commercial café and restaurant kitchen in the urban centre of Kampala, serving approximately 30–80 patrons per day. The restaurant mainly serves lunch using charcoal stoves and improved cookstoves, with charcoal as the primary fuel.

Baseline logger monitoring ran from 4 to 20 November 2025 at all three sites. During this phase, each kitchen operated using its usual fuels and appliances, including firewood and charcoal. A transition period from 20 November to 3 December was used for electrical inspection and upgrades, appliance procurement and installation, enumerator training and supervised staff practice. Intervention monitoring ran from 5 to 24 December at Site A, 12 December 2025 to 7 January 2026 at Site B, and 4 to 23 December at Site C.

All monitoring equipment was procured and pre-tested before field deployment to confirm recording and data retrieval. Electrical inspection, rewiring or upgrades were undertaken where required. Kitchen staff received hands-on training in the safe and effective use of electric pressure cookers (EPCs) over four days of supervised practice before intervention monitoring began.

**Figure 4:**
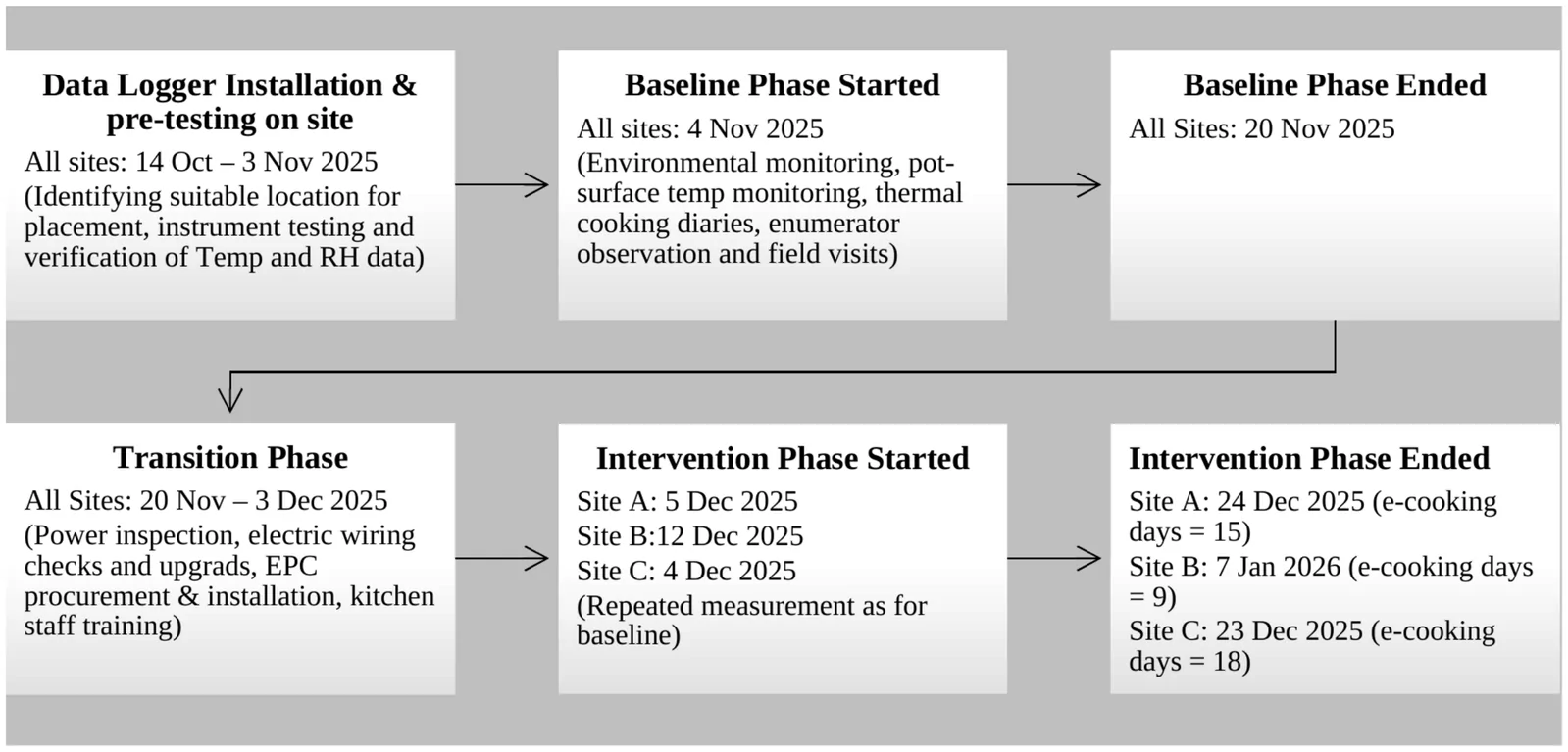
Timeline of project activities and phases.

During the intervention phase, ECO QUICKPOT EPCs (Eco Stoves Uganda, Kampala, Uganda) were introduced at each site (Site A: 2 × 45 L; Site B: 2 × 75 L plus 1 × 55 L; Site C: 2 × 21 L). An active e-cooking day was operationally defined as a day when cooking was done using electric appliances. The Site B start was delayed because the first two appliances were insufficient for the larger cooking volumes and an additional unit was required. Figure 4 summarises the project timeline and study phases.

All recorded meals on active days represented electric cooking only. Biomass use and stove stacking were not systematically recorded throughout the intervention, so concurrent or unreported combustion cooking cannot be analysed. Meal, fuel, workload and subjective response data were not collected on non-active intervention days, which cannot be retrospectively classified as outage, mixed-fuel, biomass cooking or no cooking days.

### 3.2. Data Collection and Analysis

Environmental monitoring used Tinytag Ultra 2 TGU-4500 temperature–relative humidity loggers (Gemini Data Loggers (UK) Ltd., Chichester, UK, and procured through Gemini Data Loggers SA (Pty) Ltd., Cape Town, South Africa) recording at five-minute intervals. The manufacturer specifies a temperature range of −25 to 85 °C, temperature resolution of 0.01 °C or better, a relative humidity range of 0–95% and nominal relative humidity accuracy of ±3% at 25 °C [43]. Equipment was procured, calibrated and pre-tested before deployment. Pot surface temperatures were recorded with handheld infrared thermometers at a standardised midpoint during active cooking, using three handheld infrared thermometers supplied by Capture Electronics Ltd. (Kampala, Uganda).

At each site, one logger was positioned near the cooking area, one inside the kitchen away from the principal stove, and one immediately outside or near the kitchen threshold. The indoor ambient and near-kitchen outdoor loggers were installed approximately 1.5 m above floor level. The near-stove logger was installed approximately 1 m above the stove surface to characterise the local cooking zone. Loggers were positioned out of direct solar radiation and were not fitted with radiant shields. Hourly Kampala weather station temperature and relative humidity provided the regional atmospheric comparator.

Logger timestamps and five-minute intervals were checked within the retained dates; no internal gaps were identified. Temperature readings were retained throughout. Exact relative humidity values of 0% and 100% were treated as sensor boundary values and excluded from Heat Index calculation while retaining the corresponding temperature observation. A stricter sensitivity analysis excluded every relative humidity observation above the manufacturer’s 95% range. Boundary values were concentrated in the Site C near-stove intervention series and the Site A outside intervention series.

Heat Index (HI) was calculated from temperature and relative humidity using the NOAA Rothfusz regression:

HI = −42.379 + 2.04901523T + 10.14333127RH − 0.22475541TRH  − 0.00683783T^2^ − 0.05481717RH^2^ + 0.00122874T^2^RH  + 0.00085282TRH^2^ − 0.00000199T^2^RH^2^

where

- HI = Heat Index (°F)
- T = air temperature (°F)
- RH = relative humidity (%)

For temperatures below 26.7 °C (80 °F), the Heat Index was set equal to measured air temperature, in line with the NOAA guidance, and NOAA-specified low- and high-humidity adjustments were applied [44]. Heat Index is used here as a comparative temperature–humidity screening proxy. It does not include mean radiant temperature, air velocity, clothing or metabolic workload and is therefore not treated as an occupational compliance metric or a direct measure of physiological heat strain. Wet bulb globe temperature, globe temperature and air speed measurements were not available.

Structured cooking diaries recorded meal type, dishes prepared, appliance used, cooking start and end times, people served and concurrent kitchen activities on baseline and documented active e-cooking days. Cooking duration was derived from recorded start and end times. Diaries were available for 15 baseline days at each site, 15 active days at Site A, nine active days at Site B and 18 active days at Site C. Meal-specific availability was lower where a meal was not prepared. The diary also captured repeated, time-stamped subjective assessments during cooking using the ordinal scales below. 
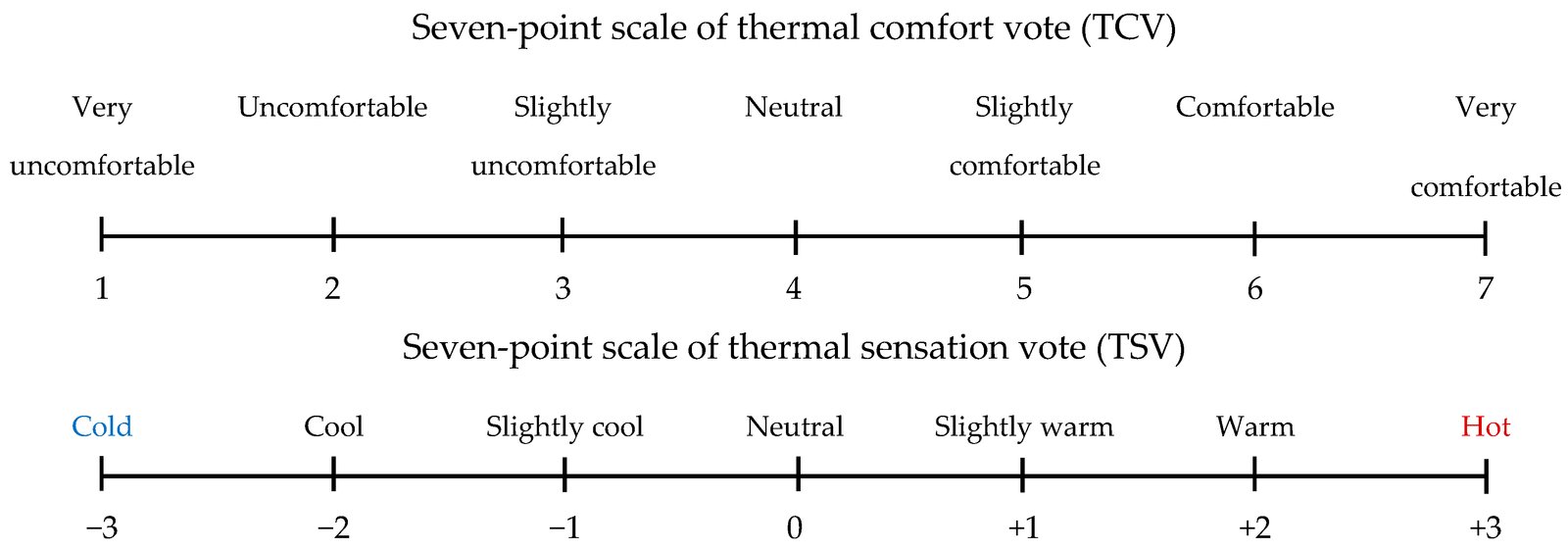


Thermal sensation votes ranged from −3 (cold) to +3 (hot), while thermal comfort votes ranged from 1 (very uncomfortable) to 7 (very comfortable). These field prompts were adapted from seven-point thermal environment assessment approaches used in kitchen and occupational research [15,21]. They are interpreted as repeated descriptive self-reports rather than diagnostic scales. Enumerator presence, recent staff training, novelty of the appliances and perceived project expectations may have influenced responses.

Enumerators maintained field notes and structured observational records to document task switching, movement, breaks, visible coping practices and known operational incidents. These records were used to interpret illustrative day profiles and to check diary timings against logger patterns. However, they did not provide a complete day-by-day register of concurrent biomass use, ventilation status, energy consumption, fuel mass, outages or stove stacking.

Five-minute data were summarised using hourly profiles and day-level statistics. The primary temperature comparison used the following active-day sets, comparing 17 baseline days with documented active e-cooking days (Site A *n* = 15; Site B *n* = 9; Site C *n* = 18). Day, rather than each five-minute reading, was the inferential unit to avoid treating serially autocorrelated measurements as independent. Mean daily temperature, Heat Index, ΔT and ΔHI were compared using two-sided unpaired Welch tests. Mean differences, site-specific standard deviations, 95% confidence intervals and *p*-values are reported. These exploratory tests describe within-site contrasts and are not pooled population-level estimates.

Meal-level cooking duration was summarised unadjusted and as minutes per 100 people served: (cooking minutes ÷ people served) × 100. Baseline and active use values were compared using unpaired Welch tests. Cooking duration was not entered as a covariate in the environmental analysis because reduced duration is a plausible pathway through which e-cooking could alter cumulative exposure. As a secondary implementation period sensitivity analysis, baseline environmental metrics were compared with all 20 weather-matched intervention period logger days at each site, regardless of registered active status. Meal, fuel, outage, stove stacking, workload and subjective response data were unavailable on non-active days. A multivariable mixed model was not fitted because there were only three heterogeneous sites and no complete daily measures of fuel use, ventilation status, stove stacking or workload on non-active days. Data visualisations were prepared using Matplotlib version 3.10.8.

The study was conducted in line with Makerere University’s ethical best practices. Participation was voluntary and based on informed consent, and confidentiality assurances were provided throughout. Kitchens were equipped in accordance with safety requirements, and participants received training on safe appliance use and emergency procedures. Enumerators also carried out regular equipment checks during fieldwork.

The study has several limitations. It used three purposively selected case study kitchens, without a parallel control, randomised intervention timing or crossover. Sequential phase differences may therefore reflect weather, building thermal response, workload, ventilation behaviour or other unmeasured temporal factors as well as cooking technology. Weather-adjusted ΔT and ΔHI reduce the influence of regional atmospheric variation but do not remove thermal inertia, local microclimate or seasonality. The primary analysis selects documented active use days. Continuous environmental data permitted an all-days sensitivity analysis, but meal, fuel, outage, stove stacking, ventilation and subjective response data were unavailable on non-active days. The study therefore cannot estimate average implementation effectiveness across the complete post-installation period. Active use records are assumed to represent exclusively electric cooking for recorded meals, although unreported combustion cooking cannot be ruled out. The study did not measure mean radiant temperature, air velocity, WBGT, physiological strain, baseline fuel mass or a meal-level energy balance.

## 4. Results and Discussion

### 4.1. Observed Cooking Practices Across Baseline and Active E-Cooking Days

**Figure 5:**
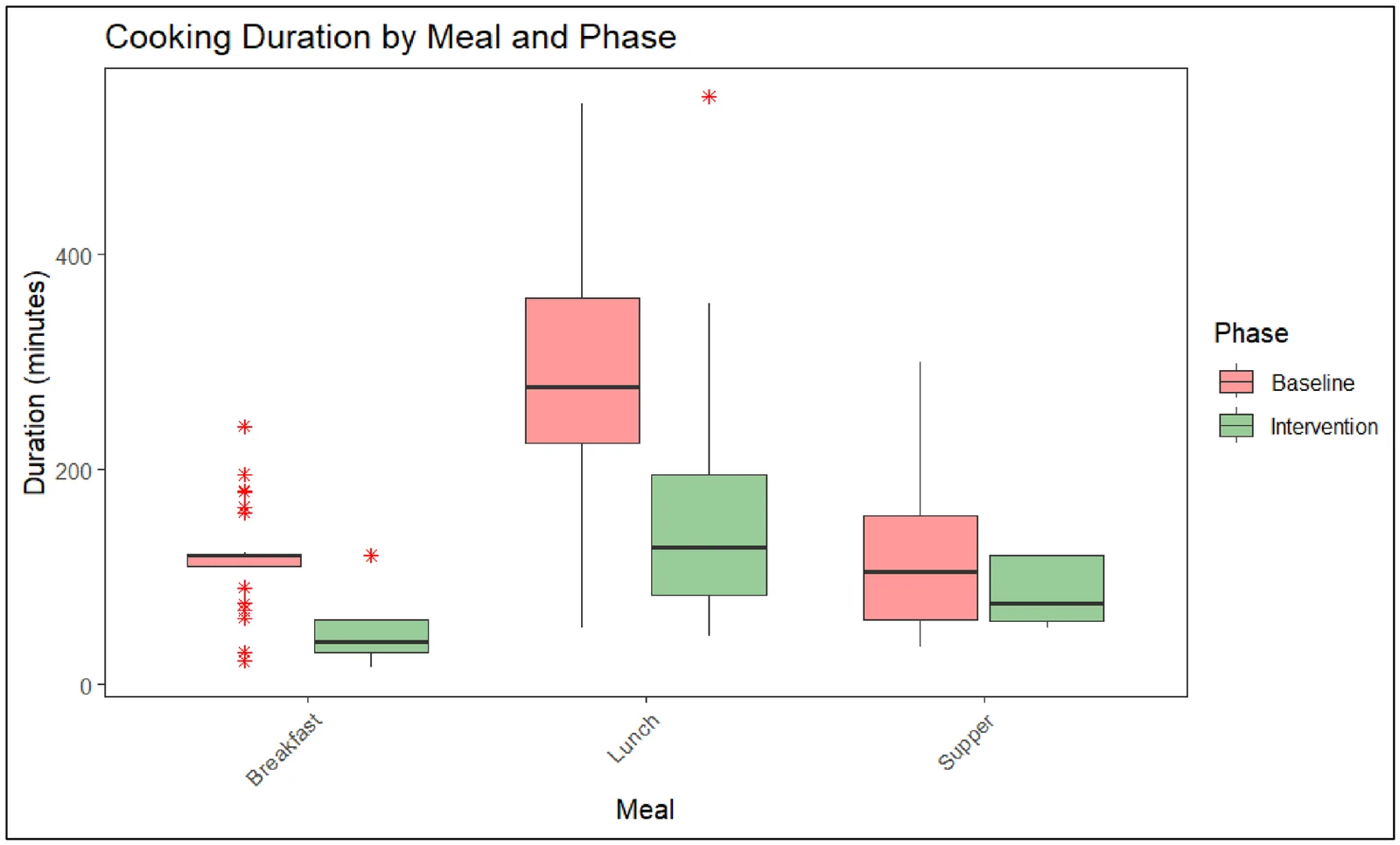
Cooking duration by meal and phase across the three sites. * denote observations beyond 1.5 times the interquartile range.

The cooking analysis concerns documented active e-cooking meals rather than every day after appliance installation. Uptake of active e-cooking days within the wider intervention phase was approximately 75% at Site A, 33% at Site B, and 90% at Site C. As shown in panel (a) of Table 2 and Figure 5, raw cooking durations were generally shorter, but Sites A and C also served fewer people. Panel (b) of Table 2 therefore adds a workload-normalised comparison. Expressing cooking duration per 100 people served tests whether the shorter recorded cooking times remained after accounting for meal volume. This sensitivity analysis complements the unadjusted durations in Table 2 without treating cooking time as a covariate in the thermal analysis, since shorter cooking duration may itself reduce cumulative exposure.

At Site A, raw mean duration fell from 113.7 to 35.0 min for breakfast and from 264.5 to 121.8 min for lunch (both *p* < 0.001). However, the mean number of people served fell from approximately 50 to 20 per meal. After normalisation by people served, neither the breakfast nor lunch difference was statistically detectable.

Site B had the most comparable meal volumes across phases. Analysis using recorded start and end times showed that raw mean duration fell from 128.0 to 67.7 min for breakfast (*p* = 0.002) and from 203.3 to 96.4 min for lunch (*p* = 0.010), while the supper difference was uncertain. After normalisation, breakfast remained 19.9 min per 100 people lower (95% CI −36.4 to −3.3; *p* = 0.021) and lunch 33.8 min per 100 people lower (−66.4 to −1.1; *p* = 0.043).

Site C prepared lunch only. Raw mean duration fell from 370.8 to 204.8 min (*p* < 0.001), while the mean number of people served fell from 64.7 to 27.2. The normalised difference was not statistically detectable (−87.4 min per 100 people; 95% CI −423.6 to 248.8; *p* = 0.597).

Workload normalisation materially qualifies the raw duration results. The reductions at Sites A and C cannot be separated from lower meal volumes. Site B retained lower minutes per 100 people for breakfast and lunch, which is consistent with faster cooking under active use conditions, although learning, altered workflow, observer presence and changes in work pace remain possible contributors.

### 4.2. Thermal Environment Across Baseline and Active E-Cooking Days

Daily temperature and relative humidity were compared between baseline logger days and documented active e-cooking days across the three logger locations (Site A *n* = 15; Site B *n* = 9; Site C *n* = 18). Panel (a) of Table 3 presents descriptive temperature summaries. Panel (b) of Table 3 supplements this by reporting day-level variability and inferential uncertainty for the near-stove comparison. It reports the standard deviation within each phase and the 95% confidence interval for the near-stove phase difference, using day rather than the five-minute logger reading as the inferential unit.

Across all three sites, baseline monitoring showed a clear spatial temperature gradient within the cooking environment, with the highest temperatures recorded near the cookstove, lower temperatures in the wider kitchen, and the lowest values outside the kitchen. This pattern was most pronounced at Sites A and C, where the near-cookstove logger captured substantially higher daily mean and daily maximum temperatures than the other logger locations. At Site A, for example, the near-cookstove logger recorded a baseline daily mean of 31.3 °C and a mean daily maximum of 47.0 °C, with an absolute peak of 59.7 °C. Site C showed a similar, though less extreme, pattern, with an absolute near-cookstove peak of 43.7 °C.

On documented active e-cooking days, daily mean temperatures were lower across the monitored kitchen environment, with the largest contrasts near the stove. Site A showed the largest difference, with near-stove daily mean temperature 5.70 °C lower and mean daily maximum 18.21 °C lower. Site C recorded a 4.05 °C lower near-stove daily mean. These differences should be interpreted alongside the lower meal volumes at both sites. Site B showed a smaller near-stove daily mean difference of 1.64 °C.

The day-level analysis in panel (b) of Table 3 corroborates the descriptive pattern in panel (a). At all three sites, the 95% confidence interval for the baseline-to-active phase difference lay below zero (Site A: −6.81 to −4.50 °C; Site B: −2.46 to −0.82 °C; Site C: −5.66 to −2.44 °C), and the contrasts were statistically detectable (*p* = 0.001 at Site B and *p* < 0.001 at Sites A and C). The lower near-stove means therefore remained evident when the five-minute observations were aggregated into daily summaries. The magnitude and variability differed between sites: Site A had the largest mean contrast, Site B the smallest, and Site C the greatest active-day variability (SD 2.73 °C) and widest confidence interval.

Lower temperatures were also observed away from the immediate cooking zone. Daily mean indoor ambient temperature was 4.27 °C lower at Site A, 2.08 °C lower at Site B and 2.00 °C lower at Site C. The magnitude varied by site and may reflect other factors such as kitchen layout, ventilation, activity intensity, meal volume, cooking duration and intervention implementation.

Relative humidity was interpreted jointly with temperature because saturation vapour pressure falls as air cools, so relative humidity can rise even when absolute moisture is unchanged. At the indoor ambient position, mean daily relative humidity changed from 54.1% to 64.5% at Site A, 62.1% to 61.8% at Site B and 56.2% to 59.5% at Site C. Higher relative humidity on active use days therefore does not by itself indicate deterioration; it may reflect cooler air, cooking moisture, ventilation or sensor exposure to steam. Table 4 presents the corresponding indoor ambient summaries.

To account for day-to-day and seasonal variation in weather conditions between the baseline period in November and the intervention period in December, excess temperature relative to background outdoor conditions (ΔT) was also calculated. ΔT was defined as the difference between the hourly mean logger temperature and the corresponding hourly air temperature from Kampala weather station data. This provides a weather-adjusted measure of thermal uplift within the kitchen environment, indicating how much warmer the monitored location was than prevailing outdoor conditions at the same hour. ΔT characterises thermal uplift relative to regional weather, but it does not isolate cooking from building response, local microclimate or other time-varying conditions.

**Figure 6:**
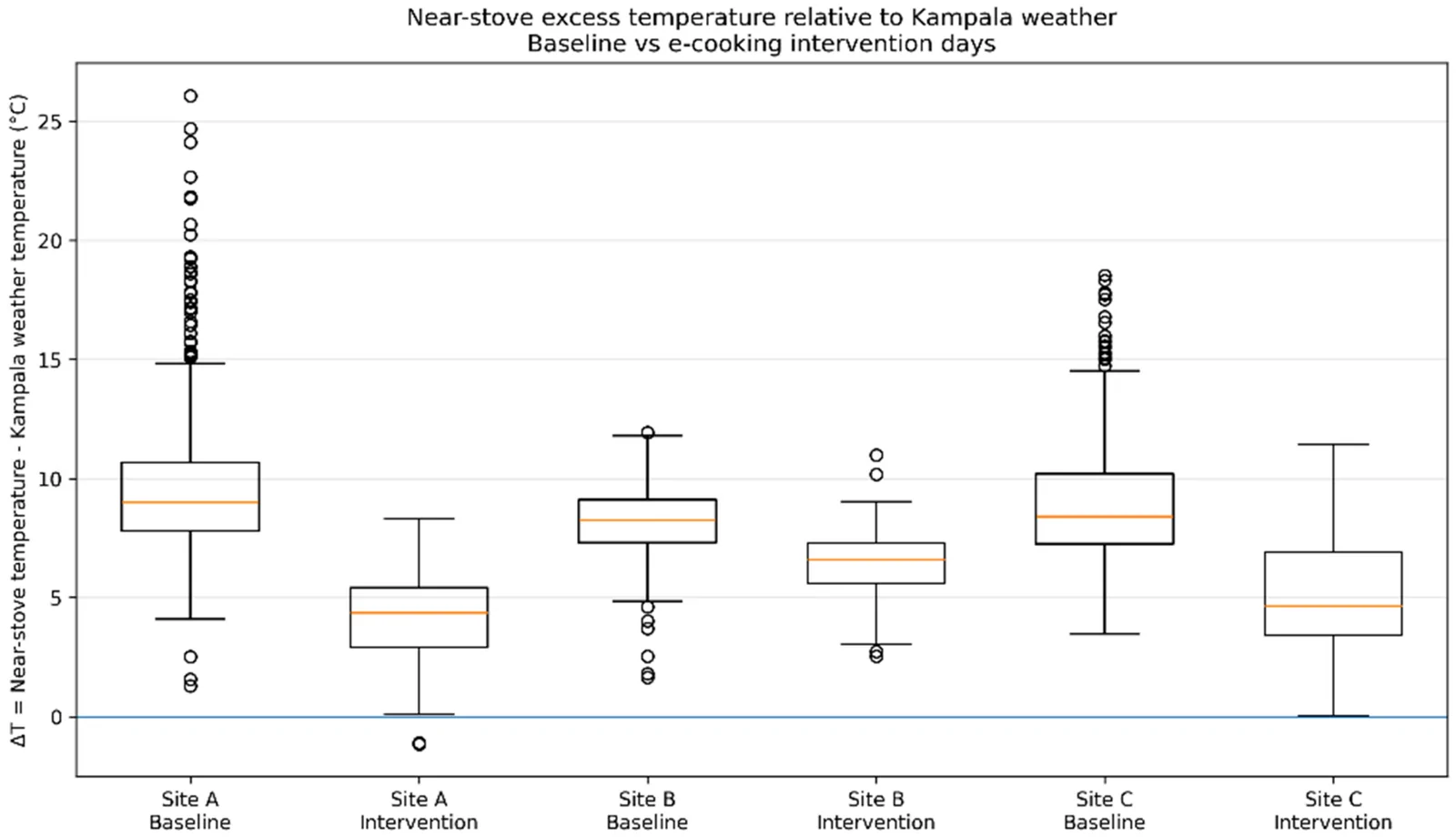
Box plot distribution of near-stove excess temperature relative to Kampala weather (ΔT) across baseline and active e-cooking days. Circles denote observations beyond 1.5 times the interquartile range; orange lines indicate medians and the blue horizontal line denotes ΔT = 0.

Figure 6 shows a consistent downward shift in near-cookstove ΔT across all three sites on active use days. Mean hourly ΔT changed from 9.90 °C at baseline to 4.22 °C on active use days at Site A, from 8.11 °C to 6.45 °C at Site B and from 9.10 °C to 5.08 °C at Site C. Thus, the monitored near-stove location showed less excess heat above the regional outdoor comparator under the observed active use conditions, with the largest contrast at Site A. These descriptive differences do not isolate the influence of cooking technology from workload, duration, ventilation or other implementation changes, all of which may also have contributed.

**Figure 7:**
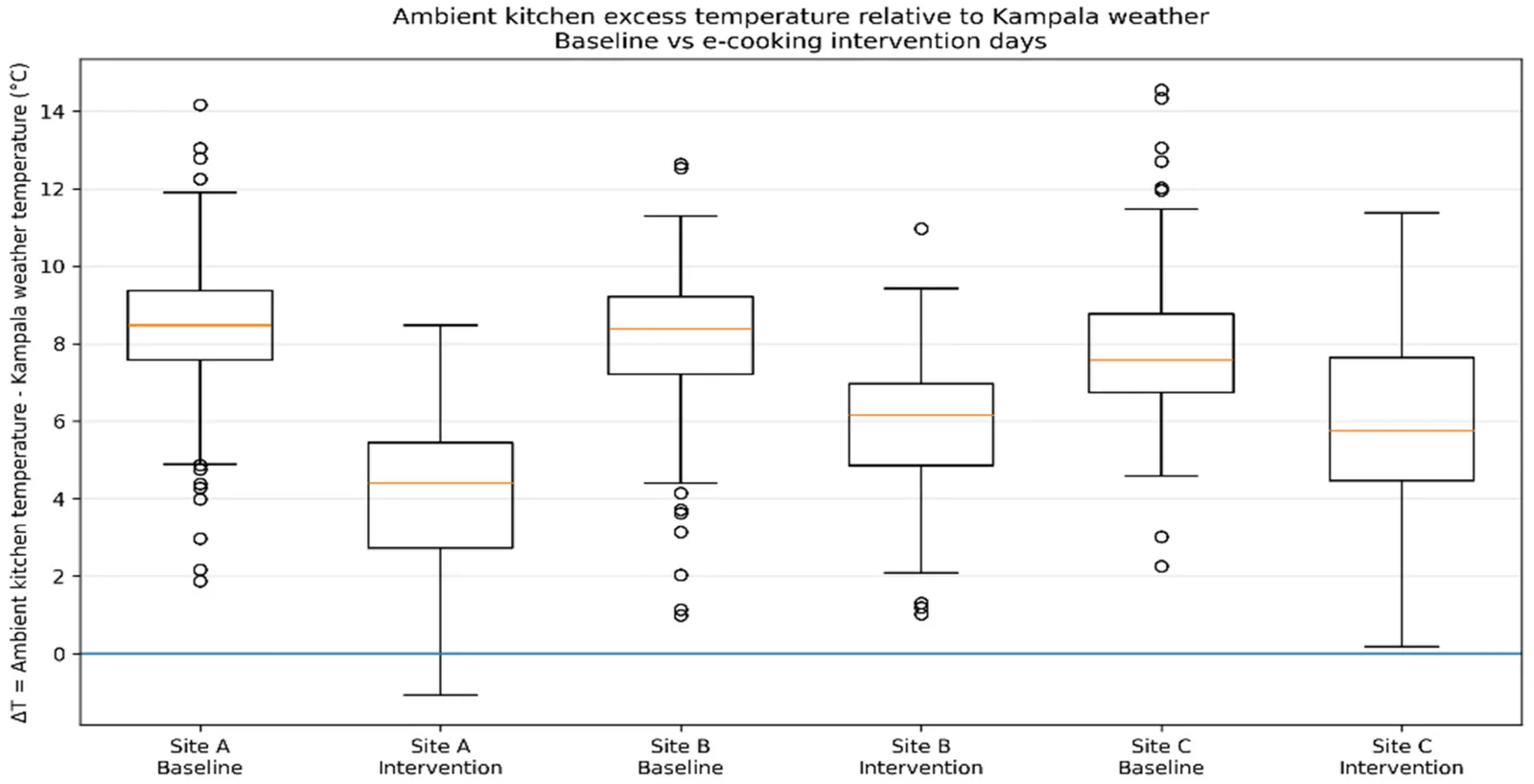
Box plot distribution of ambient kitchen excess temperature relative to Kampala weather (ΔT) across baseline and active e-cooking days. Circles denote observations beyond 1.5 times the interquartile range; orange lines indicate medians and the blue horizontal line denotes ΔT = 0.

Figure 7 shows that the wider kitchen environment was also cooler relative to background weather on active use days, although the difference was smaller than at the stove. Mean hourly indoor ambient ΔT fell from 8.46 °C to 4.22 °C at Site A, from 8.11 °C to 5.89 °C at Site B and from 7.81 °C to 5.84 °C at Site C. Weather-adjusted differences were also evident immediately outside the kitchens, although these patterns were less pronounced and more sensitive to local conditions. The profiles indicate lower stove-edge and wider room thermal uplift under the observed active use conditions; however, they do not isolate cooking technology from workload, duration, ventilation or implementation change.

### 4.3. Apparent Heat Burden and Exposure Risk

**Figure 8:**
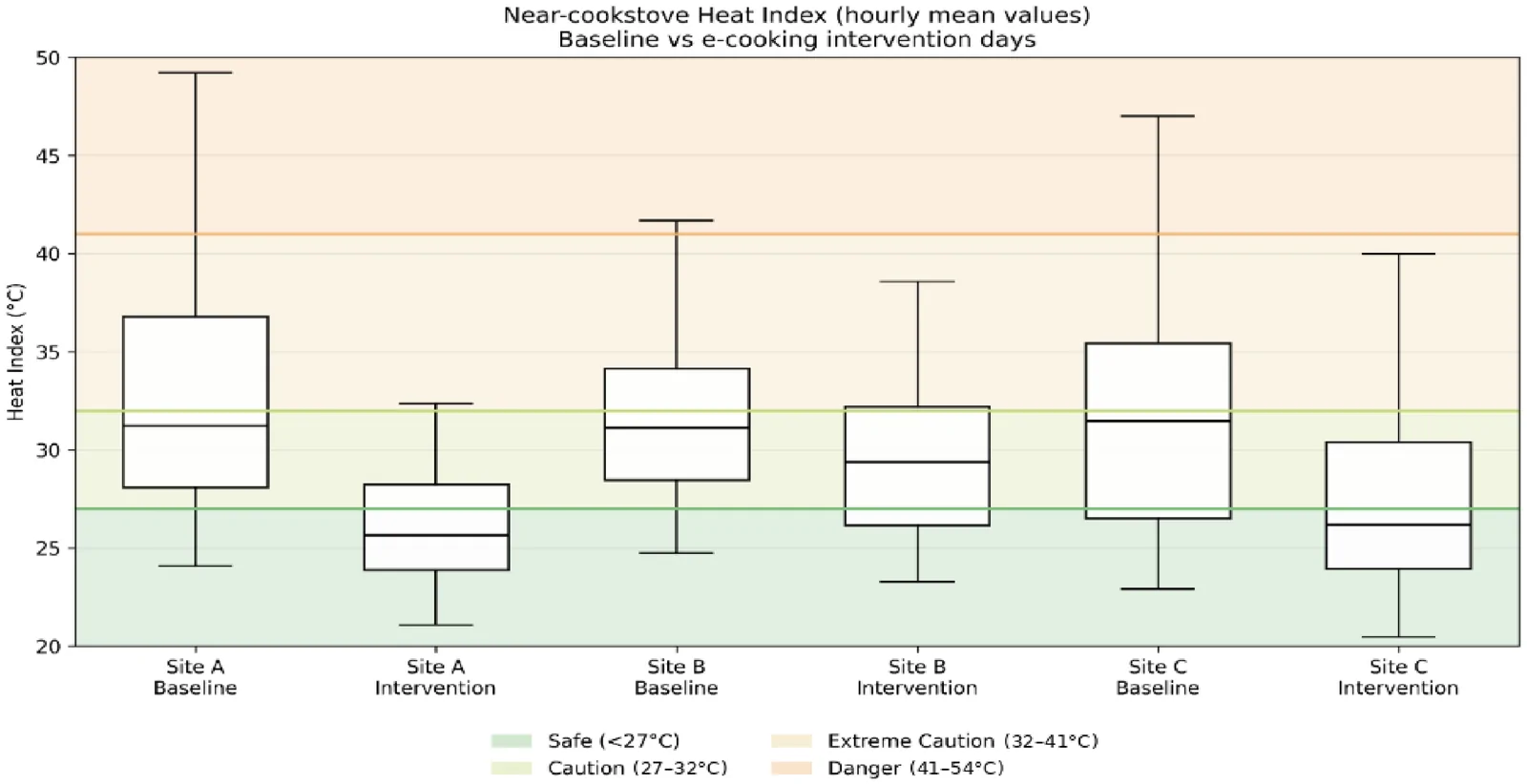
Near-cookstove Heat Index (hourly mean) on baseline and active e-cooking days across the three sites. Horizontal coloured lines and shaded bands denote the Heat Index screening thresholds and categories shown in the legend; box centre lines show medians.

**Figure 9:**
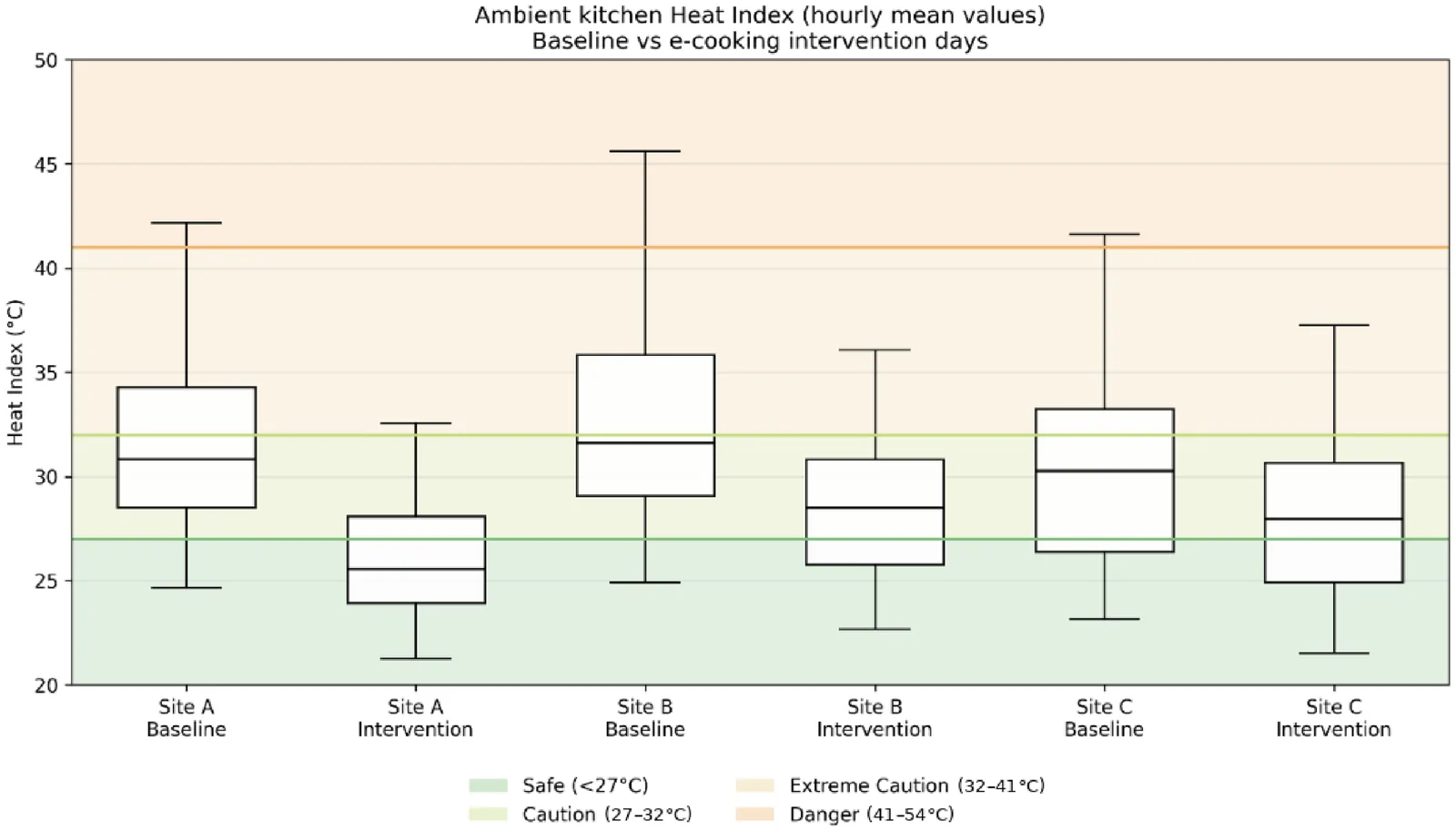
Ambient kitchen Heat Index (hourly mean) on baseline and active e-cooking days across the three sites. Horizontal coloured lines and shaded bands denote the Heat Index screening thresholds and categories shown in the legend; box centre lines show medians.

Heat Index was used to characterise the combined temperature–humidity contribution to apparent heat burden. Figure 8 and Figure 9 present the phase-specific hourly descriptive profiles for the near-stove and indoor ambient positions. Hourly aggregation reduces the influence of very short-lived spikes, particularly near the stove. Exact relative humidity boundary readings of 0% and 100% were excluded from Heat Index calculation only, with the corresponding temperature observations retained. It is important to note that Heat Index does not capture radiant heat or air movement and is not a definitive occupational exposure measure, but provides a screening indication of apparent heat burden in the monitored environment.

Across all three sites, the hourly descriptive profiles shifted downward on active use days at both indoor logger locations. Mean hourly near-stove Heat Index changed from 33.26 °C to 26.00 °C at Site A, 31.35 °C to 29.55 °C at Site B and 31.71 °C to 27.50 °C at Site C. In each case, the lower medians and narrower interquartile ranges are consistent with lower apparent heat burden and reduced hourly variability.

**Figure 10:**
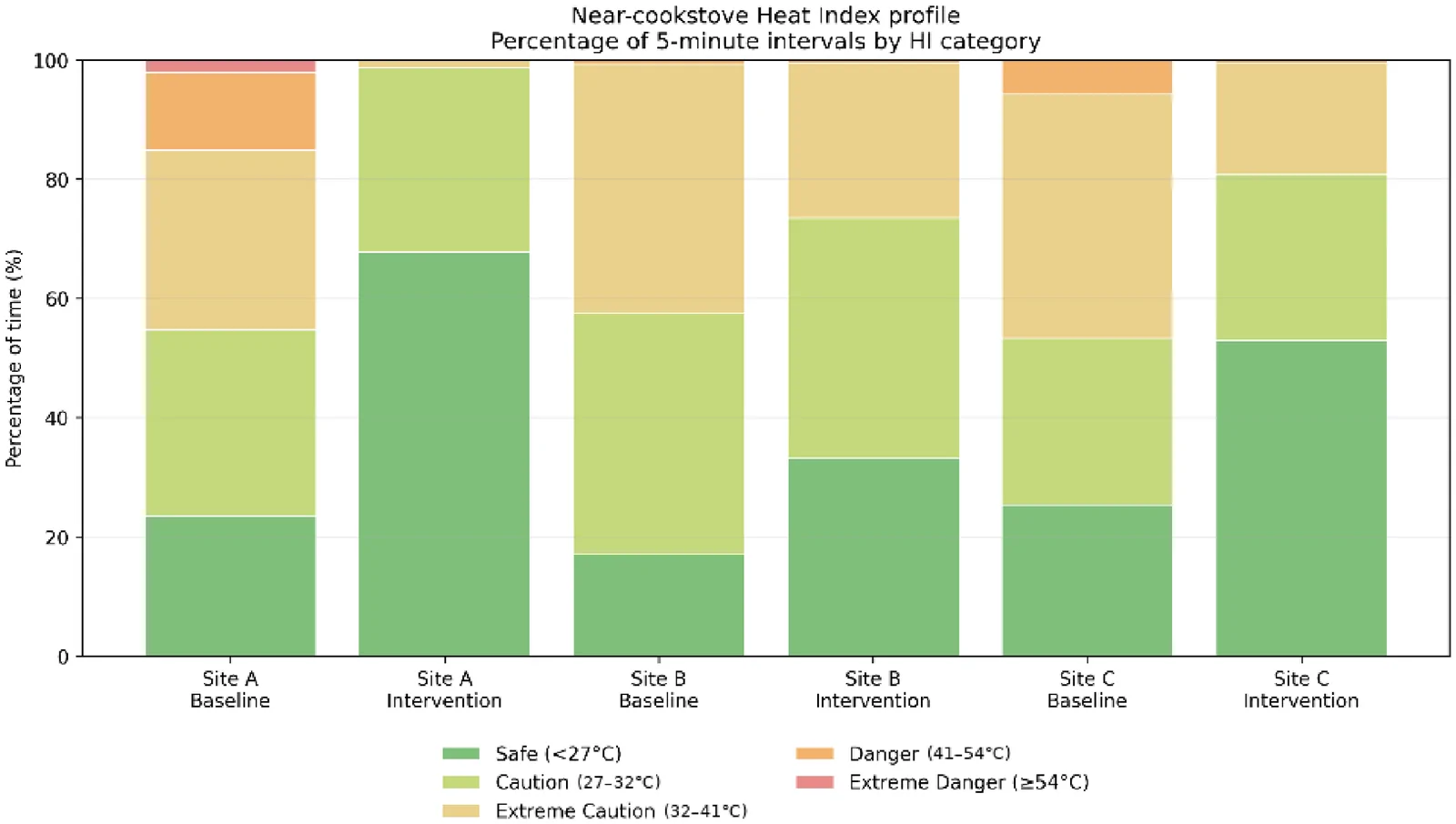
Descriptive percentage distribution of near-stove Heat Index screening categories across baseline and active e-cooking days.

**Figure 11:**
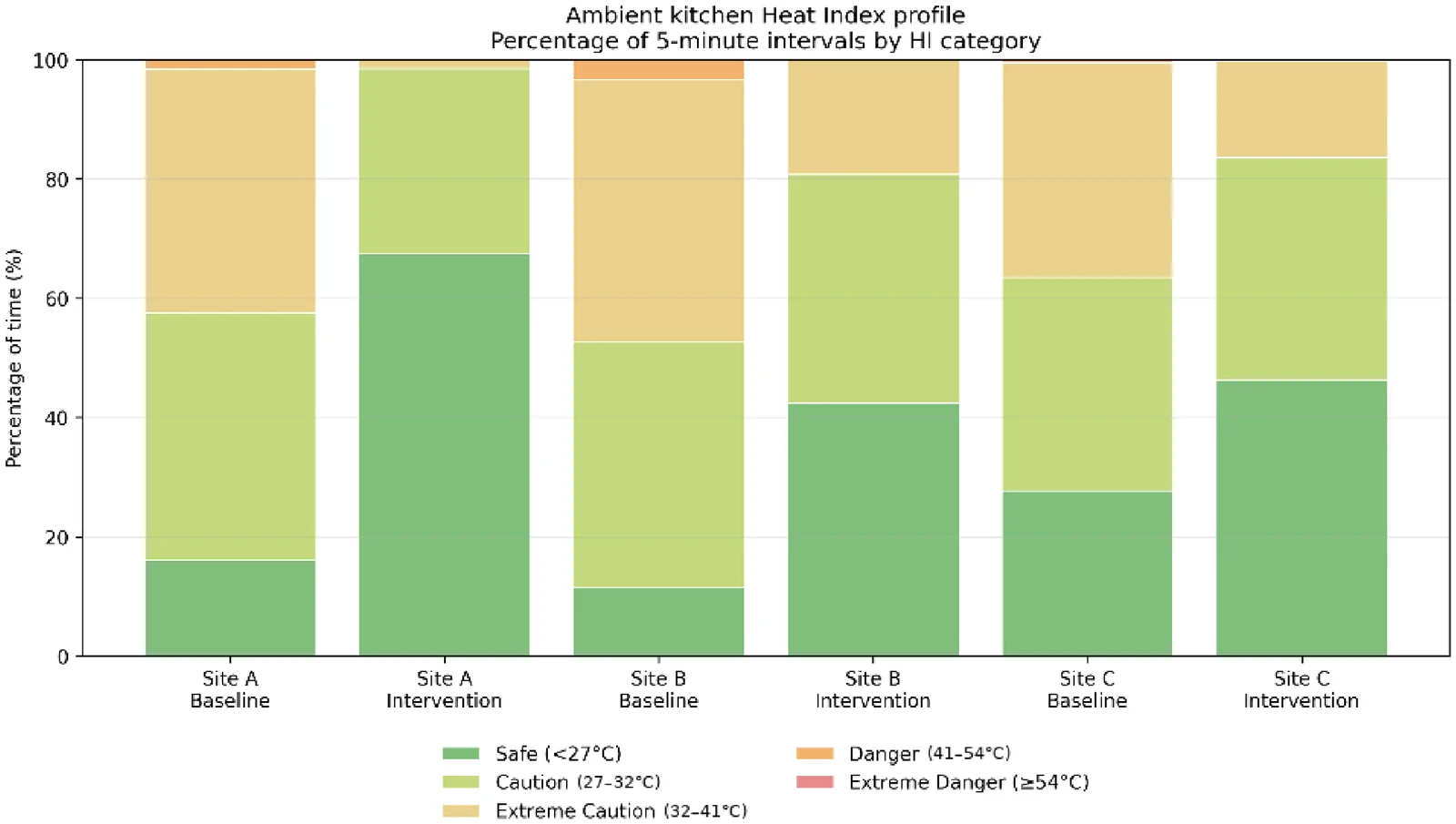
Descriptive percentage distribution of indoor ambient Heat Index screening categories across baseline and active e-cooking days.

The descriptive Heat Index risk profiles based on five-minute readings show the same directional pattern (Figure 10 and Figure 11). Near the stove, the proportion of time in the Safe category increased from 23.5% to 67.8% at Site A, from 17.0% to 33.3% at Site B, and from 25.4% to 53.0% at Site C. These category summaries are screening descriptions rather than estimates of occupational compliance, because Heat Index omits radiant heat, air velocity, clothing and metabolic workload. At Site C, Danger exposure fell from 5.7% to 0.3%. Site B showed a smaller but still meaningful reduction, with the share of time in Extreme Caution falling from 41.9% to 25.9%.

A similar pattern was observed in the ambient kitchen environment. At Site A, the share of time in the Safe category rose from 16.2% at baseline to 67.6% on active e-cooking days, while combined Extreme Caution and Danger exposure fell from 42.3% to 1.4%. Site B also showed improvement, with Safe exposure increasing from 11.5% to 42.5% and Danger exposure disappearing. At Site C, Safe exposure rose from 27.7% to 46.3%, while Extreme Caution fell from 35.9% to 16.0%.

To reduce the influence of seasonal and day-to-day variation in regional weather, ΔHI was calculated as logger Heat Index minus concurrent Kampala weather station Heat Index. Lower ΔHI on active use days indicates lower excess apparent heat relative to the regional comparator. This adjustment does not remove building thermal inertia, site microclimate or other unmeasured time-varying factors that may still affect heat exposure.

**Figure 12:**
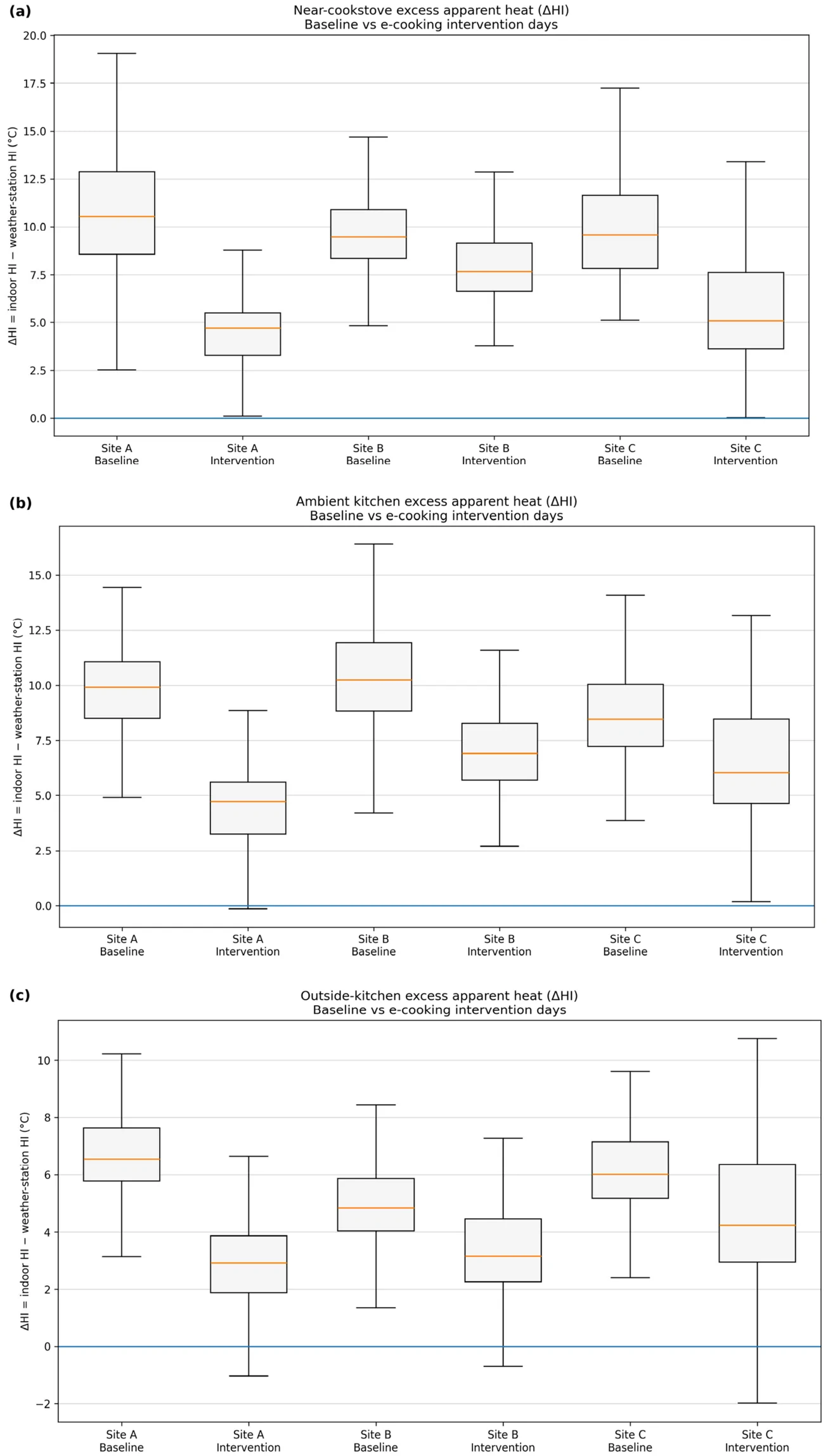
Distribution of hourly excess apparent heat (ΔHI) relative to Kampala weather station conditions across baseline and active e-cooking days for the (**a**) near-cookstove, (**b**) ambient kitchen, and (**c**) outside logger locations. Orange lines indicate medians and blue horizontal lines denote ΔHI = 0.

Figure 12 shows lower mean hourly ΔHI on active use days, especially near the stove. Near-stove ΔHI was 7.11 °C lower at Site A, 1.65 °C lower at Site B and 4.36 °C lower at Site C. The corresponding indoor ambient differences were −5.26 °C, −3.91 °C and −2.33 °C. These are weather-relative environmental contrasts rather than physiological heat strain effects.

Taken together, the environmental data show lower apparent heat burden and less time in higher Heat Index screening bands under documented active use conditions, with the largest contrasts close to the stove. Whilst these associations may have resulted from the e-cooking intervention, they should not be interpreted as appliance-only or causal effects because workload, cooking duration, ventilation, calendar time and implementation conditions also differed.

Table 5 complements the hourly ΔHI profiles by reporting day-level variability and uncertainty at the near-stove position, where the largest thermal contrasts were observed. Each monitored day is treated as the inferential unit, avoiding the assumption that serial five-minute observations are independent.

The day-level estimates confirm lower weather-adjusted apparent heat at the near-stove position at all three sites. The contrast was largest at Site A and smallest at Site B, consistent with the hourly profiles. These are within-site phase associations; weather adjustment reduces regional atmospheric differences but cannot remove workload, ventilation, building response or other temporal changes.

Table 6 tests the sensitivity of the environmental pattern to the selection of documented active e-cooking days by comparing the active use estimate with all 20 weather-matched intervention period logger days at each site. This analysis can assess whether the direction and approximate magnitude depend heavily on active-day selection, but it cannot identify the fuels used, cooking activity, outages, stove stacking, workload or workers’ thermal experience on non-active days.

The all-days changes were close to the active use estimates at each site. This indicates that the direction and approximate magnitude of the December environmental contrast were not produced solely by excluding non-active days. However, it does not show that e-cooking was used on every intervention day or quantify real-world uptake.

The Site C near-stove intervention series contained 414 exact 0% or 100% relative humidity readings among 5029 retained active-day observations (8.2%); 648 readings exceeded the manufacturer’s 95% range. Excluding every reading above 95% changed the Site C near-stove hourly Heat Index difference from −4.19 °C to −3.86 °C. The direction and overall interpretation were unchanged.

### 4.4. Integrating Time Series, Diaries and Workers’ Thermal Experience

To examine how heat exposure varied across the day, diurnal time series of mean hourly excess apparent heat (ΔHI) were derived for the near-cookstove, ambient kitchen, and outside logger locations. These profiles show the typical daily rhythm of cooking-related heat amplification relative to outdoor conditions, while smoothing short-lived fluctuations and day-specific anomalies. They therefore allow clearer comparison of when thermal burden was greatest at each site, how strongly it tracked cooking activity, and how the intervention altered these temporal patterns.

**Figure 13:**
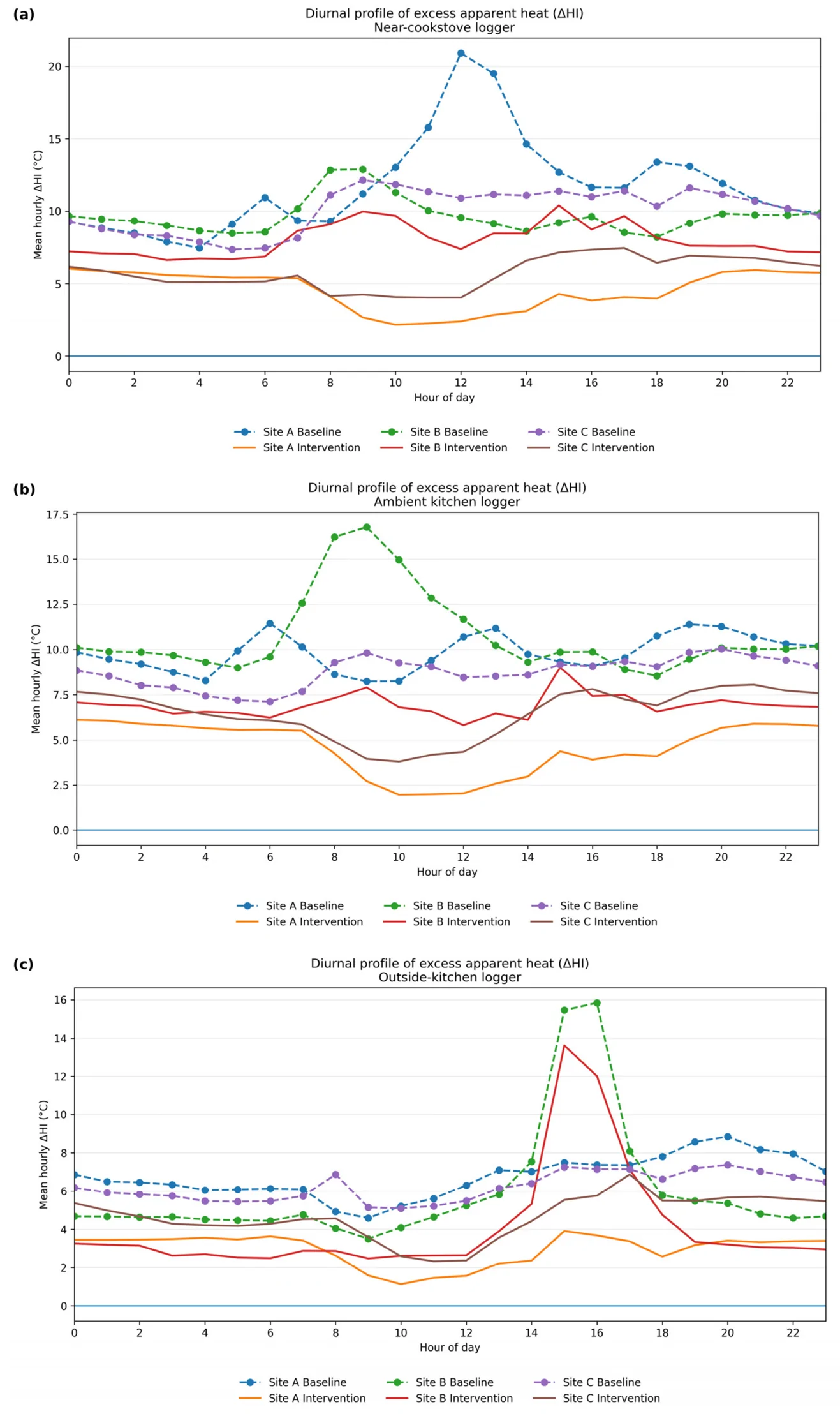
Diurnal profile of mean hourly ΔHI across baseline and active e-cooking days for the (**a**) near-cookstove, (**b**) ambient kitchen, and (**c**) outside logger locations.

Across the three sites, baseline ΔHI generally peaked during the main cooking windows identified in the diaries, particularly in the late morning and early afternoon (Figure 13). Site A showed the clearest midday amplification, while Site C showed a broader daytime elevation linked to prolonged lunch preparation. On active use days, these peaks were lower, especially at Sites A and C. At Site A, for example, mean near-stove ΔHI across 10:00–14:00 fell from 16.8 °C to 2.5 °C. The same direction was visible in the indoor ambient series, although the peaks and phase contrasts were smaller.

The outside logger showed weaker and less clearly timed diurnal variation than the two indoor logger positions. Whereas the near-cookstove and ambient kitchen loggers tracked cooking-related peaks reasonably well, the outside logger appears to capture a more mixed microclimatic signal, likely shaped by external factors such as local airflow, shading, doorway effects, and rainfall, in addition to heat spill from the kitchen.

At Site B, although e-cooking days were associated with reduced ΔHI at both the near-cookstove and ambient kitchen locations, the shift was smaller and less concentrated around a single midday peak. Several factors likely contributed to this. First, baseline cooking at Site B already relied on improved cookstoves, which likely reduced the initial heat penalty relative to the more heat-intensive baseline conditions at Sites A and C. Second, the kitchen was more spacious and better ventilated, with lower typical surface temperatures and stronger passive heat dissipation. Third, its daily routine was more continuous, with overlapping breakfast, lunch, and supper preparation rather than one or two more discrete cooking blocks. Finally, the active e-cooking period during the intervention phase at Site B was shorter and more disrupted because of delayed rollout, limited initial electrical capacity, and a power circuit incident that temporarily halted electric cooking. These operational interruptions likely weakened the contrast between baseline and active e-cooking days and help explain why the diurnal pattern for Site B appears flatter and more mixed than the clearer intervention effects seen at Sites A and C.

The quantitative HI results were further interpreted using selected day-level time series, cooking diaries, repeated thermal comfort and sensation votes, and enumerator observations. These materials help explain the timing and context of apparent heat burden and why its magnitude and pattern differed across sites.

**Figure 14:**
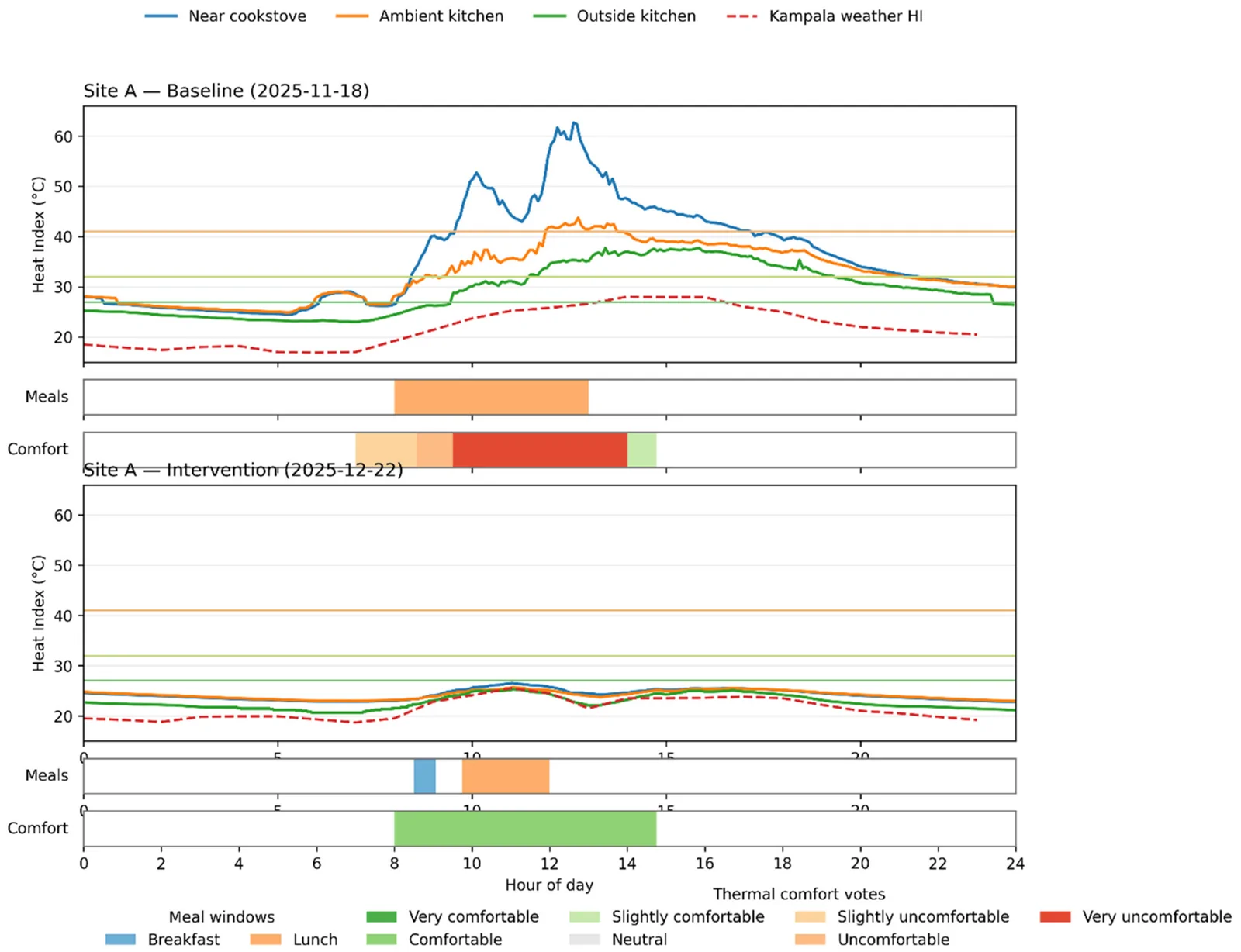
Illustrative Heat Index (HI) time series for selected baseline and active e-cooking days at Site A.

At Site A, the baseline and active e-cooking day contrast was especially pronounced (Figure 14). On the selected baseline day, near-cookstove and ambient kitchen HI rose steeply during overlapping breakfast and lunch preparation and remained elevated through much of the morning, producing a broad mid-morning to early afternoon peak. This reflected the school kitchen’s prolonged and overlapping workflow, in which breakfast ran from roughly 08:00–11:00 and lunch from about 08:00–13:00, with high stove surface temperatures ranging from 40 to 118 °C, leaving little opportunity for thermal recovery between tasks. In this semi-enclosed kitchen, passive wall openings were the main means of ventilation and heat dissipated only gradually. Diary-based thermal sensation and comfort votes tracked this pattern: reported sensation shifted into the warm-to-hot range, while comfort deteriorated towards slightly uncomfortable, uncomfortable, and very uncomfortable. Enumerator field observations similarly described the kitchen as becoming “hot and very uncomfortable” once all stoves were in use, especially around midday, when heat from the iron sheet roof compounded stove-generated heat.

For the selected active e-cooking day, both breakfast and lunch were prepared in much shorter time periods: breakfast ran for about 08:30–09:00 and lunch for roughly 09:45–12:00. Rather than one long continuous block of elevated heat, the active use day HI profile shows a lower and more interrupted cooking-related rise, followed by a quicker return towards lower levels. This coincides with the Site A summaries, in which mean breakfast duration was around 69% shorter and lunch duration around 54% shorter, while ambient kitchen temperatures were approximately 4–5 °C lower and previously common hotspots were absent. However, the selected active use day served only around 75% as many people as the selected baseline day. The observed contrast therefore coincided with documented active e-cooking and a substantially lower cooking load; it cannot be attributed to either factor alone.

**Figure 15:**
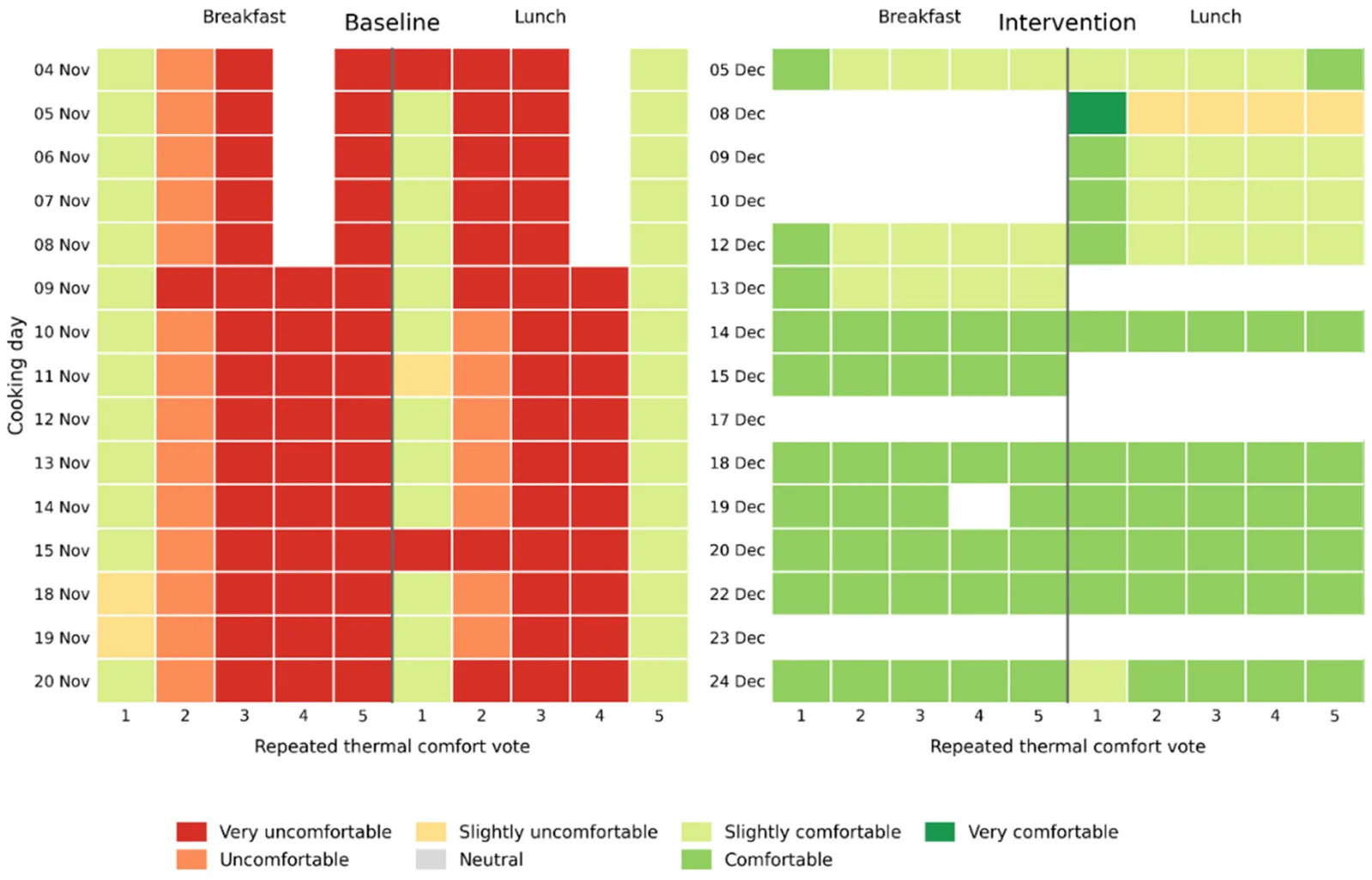
Site A thermal comfort votes across baseline and active e-cooking days.

On the selected Site A active use day, workers reported cooler sensations and more favourable comfort, while enumerators recorded fewer reactive coping behaviours than on the selected baseline day, such as removing clothing, wiping sweat, stepping outside, and taking frequent pauses. These descriptive patterns, summarised across recorded meals in Figure 15, are consistent with the environmental measurements but cannot confirm a causal subjective response.

**Figure 16:**
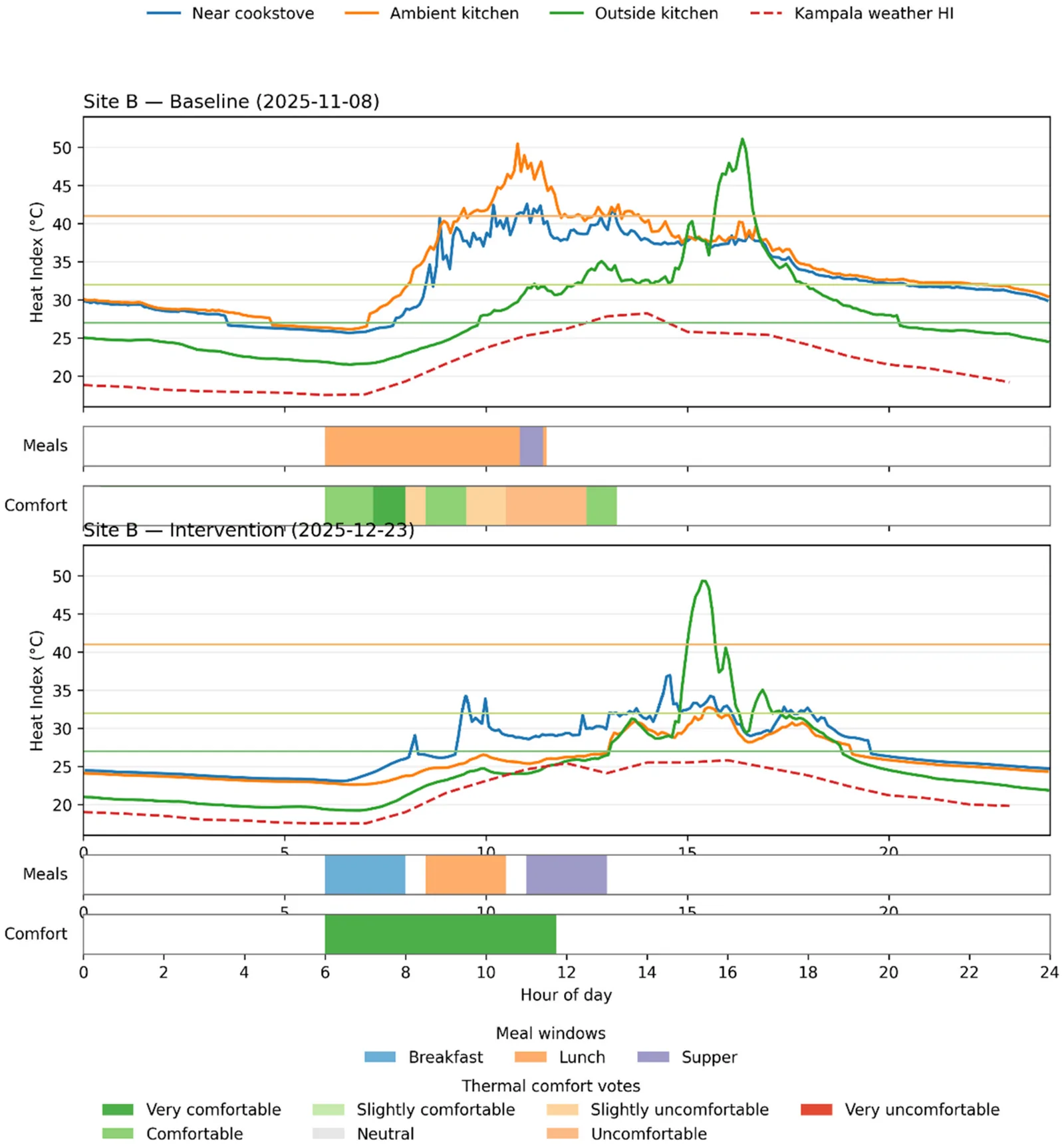
Illustrative Heat Index (HI) time series for selected baseline and active e-cooking days at Site B.

For Site B, the selected baseline and active e-cooking day shown in Figure 16 included three meals and comparatively similar meal volumes. On the baseline day, near-stove and indoor ambient Heat Index rose through the morning and remained elevated across a broad late morning to early afternoon window. The diary recorded breakfast, lunch and supper, including a 330 min firewood-cooked lunch. On the selected active use day (23 December), the recorded start and end times give 120 min for each meal. The Heat Index profile was lower and more compressed, while sensation and comfort were reported more favourably.

The more muted contrast at Site B is supported by the observational data. Enumerators described this kitchen as comparatively spacious and well ventilated, with tiled walls, several windows, large ventilation openings, and a larger overall floor area than the other sites. They noted that, on average, the thermal environment was generally tolerable and that smoke and soot from firewood were often more troublesome than heat alone, although conditions became less comfortable closer to midday and immediately beside the cooking pots. Cooking was concentrated mainly in the morning, after which food was often left to steam until serving and cooks coped by wiping sweat, stepping out of the kitchen, and in some cases removing outer clothing. These observations help explain why the Site B HI curve appears broader but flatter: the site already had lower baseline thermal intensity, stronger passive ventilation, and a workflow that spread cooking over overlapping meal preparations rather than one or two concentrated peaks. It is also important to note that at Site B, the outside-kitchen HI profile shows a recurring late afternoon spike between roughly 15:00 and 18:00 in both baseline and intervention, likely indicating the influence of external site-specific factors shaping the immediate threshold microclimate.

**Figure 17:**
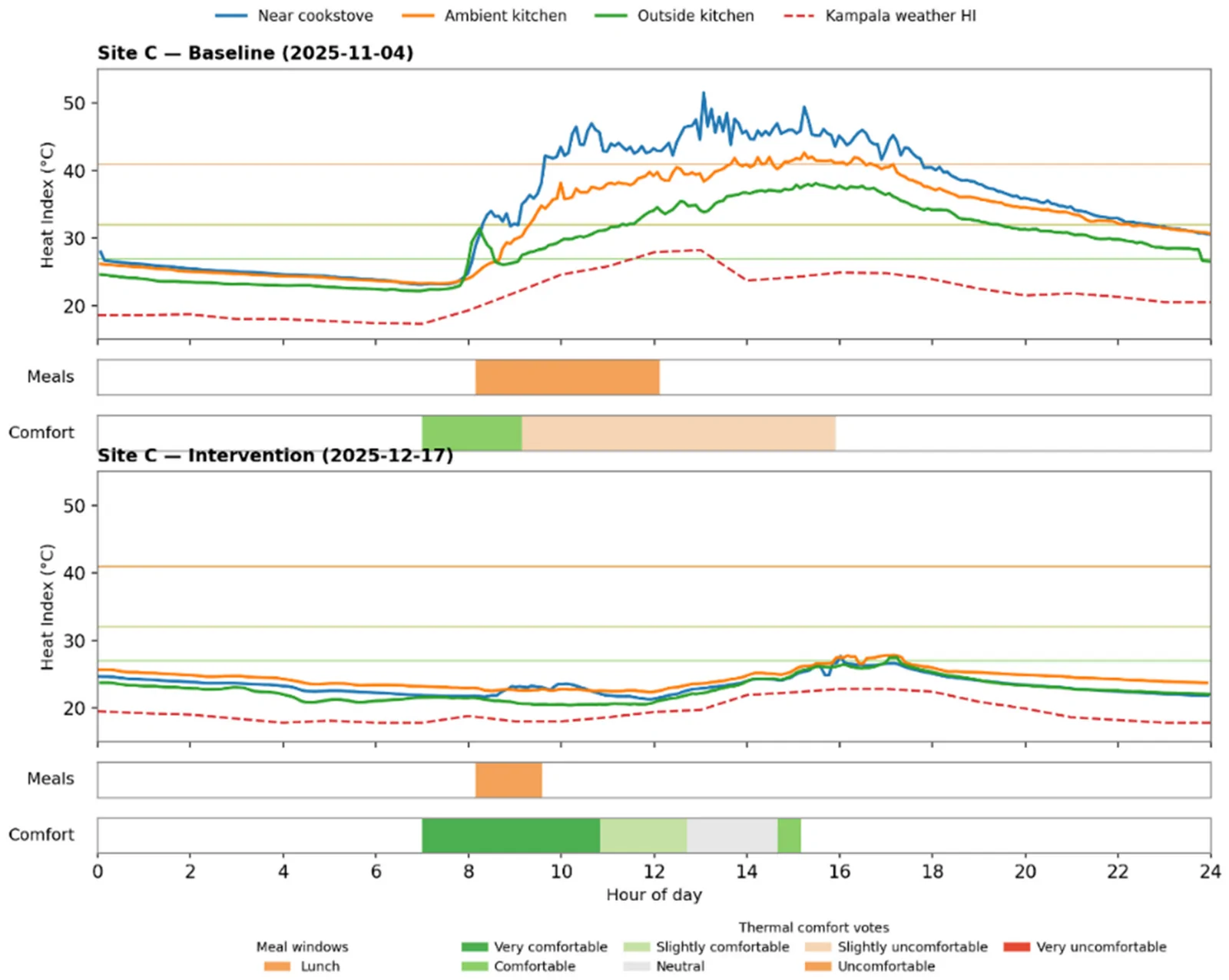
Illustrative Heat Index (HI) time series for selected baseline and active e-cooking days at Site C.

At Site C (Figure 17), baseline charcoal-based lunch preparation was prolonged and produced sustained stove edge Heat Index elevation. On the selected active use day, lunch was shorter and prepared with an EPC, although only around 60% as many people were served as on the selected baseline day. Reported comfort was more favourable, although Heat Index remained elevated beyond the recorded cooking window, consistent with preparation, holding, serving and other stove-side activity not fully captured by diary timings. The selected-day contrast therefore shows lower measured thermal exposure under active use conditions, qualified by the smaller workload and continuous commercial workflow.

### 4.5. Implications of the Heat Exposure Data for Public Health

The results contribute to public health understanding by documenting spatially uneven environmental thermal exposure in three working kitchens, consistent with the wider occupational heat literature [2,3,5]. Near-stove conditions were generally hotter than the wider kitchen, showing why a single whole-room measurement may understate the local temperature–humidity burden during stove-side tasks. These environmental data are relevant to occupational health, although they do not measure physiological strain.

Taken together, the day-level uncertainty analysis strengthens the consistency of the central temperature finding while also qualifying it. Statistical separation between phases did not depend on treating serial five-minute readings as independent observations, although the analysis does not convert the observed phase contrasts into causal effects. The higher between-day variability at Site C indicates less uniform active use conditions, while the sequential design, workload differences and other site-specific conditions remain possible contributors to all three estimates.

Documented active e-cooking days were associated with lower near-stove temperature and apparent heat burden at all three sites, with the largest contrasts at Sites A and C. Heat Index is useful for comparing temperature–humidity conditions across phases, but it does not capture radiant heat from flames, hot stove surfaces and vessels, or changes in air speed caused by ventilation [2,13]. WBGT incorporates a globe temperature component and is more closely aligned with occupational heat assessment, although interpretation would still require metabolic workload, clothing and work–rest context.

The repeated thermal comfort and sensation reports and observed coping practices provide descriptive context for the logger data and respond to calls to connect measured exposure with worker experience and workplace routines [5,20]. Workers generally reported more favourable conditions during selected active use meals, but training, appliance novelty, enumerator presence and perceived expectations may have influenced these responses. Future studies should combine air temperature and relative humidity with globe temperature, air velocity and physiological indicators such as heart rate, hydration or core temperature.

## 5. Conclusions and Future Implications

In these three Kampala case study kitchens, documented active e-cooking days were associated with lower near-stove and indoor ambient temperature and lower weather-adjusted apparent heat burden than the preceding combustion-based baseline. The magnitude varied substantially by site and was the smallest at Site B. These findings concern environmental thermal exposure and reported experience; they do not establish a reduction in physiological heat strain.

Removal of open flames and better insulation of electric pressure cookers are plausible mechanisms for lower local heat release, consistent with earlier comparisons of electric and combustion-based kitchens [15,41], while shorter cooking time may reduce cumulative exposure. The study did not measure baseline fuel mass, meal-level electrical consumption, radiant temperature or air speed, so these pathways cannot be quantified. Workload analysis also qualifies the cooking time results: after normalisation by the number of people served, reductions persisted for Site B breakfast and lunch but were not statistically detectable at Site A or C.

The scale of the observed contrast varied across sites. Site A showed the largest near-stove daily mean temperature difference (−5.70 °C), followed by Site C (−4.05 °C) and Site B (−1.64 °C). An environmental sensitivity analysis including all intervention period logger days produced near-stove ΔHI differences similar to the active use estimates, suggesting that the thermal contrast was not driven solely by excluding non-active days. Nevertheless, the sensitivity analysis cannot identify cooking activity, fuels, outages, workload or subjective experience on those days.

The study contributes rare spatially resolved, mixed-method field evidence from institutional and commercial kitchens in an urban African setting, extending earlier kitchen research [15,33,41]. It also helps move the health-focused clean cooking debate beyond an emissions-only framing towards a possible occupational heat intervention pathway through clean cooking transitions. Although the three purposive cases do not support population-level extrapolation or a standardised technology effect, their value lies in characterising local temperature–humidity conditions and relating them to detailed meal records through time-stamped cooking diaries, workers’ subjective reports of heat exposure and implementation constraints.

Several limitations affect the generalisability of the findings but also highlight clear directions for future research. The principal limitations are the three-site purposive sample, absence of a parallel control or randomised timing, short sequential monitoring periods, architectural heterogeneity and incomplete workload comparability. Weather-adjusted metrics cannot remove building thermal inertia, local microclimate, seasonality or unmeasured calendar time change. The primary analysis selects documented active use days and cannot rule out unreported combustion cooking. Meal, fuel, ventilation, outage, stove stacking and subjective response data were unavailable on non-active days. Further, the study did not measure air change rates, mean radiant temperature, air velocity, WBGT or physiological indicators, nor did it collect the data required for an energy balance.

Within these limits, the findings indicate that kitchen thermal exposure should be considered when evaluating clean cooking transitions in institutional and commercial settings [2,3,45]. Clean cooking interventions need to be assessed through their effects on heat exposure and occupational thermal comfort, in addition to emissions reduction, particularly in institutional and commercial settings where cooking-related heat is sustained, concentrated, and often poorly managed. However, the study also indicates that technological substitution by itself may be insufficient. Electricity reliability, appliance capacity, wiring, ventilation, meal volume and workflow are likely to shape whether lower local thermal exposure can be sustained in routine practice. Larger studies using parallel or phased comparison groups, continuous fuel and workload records, globe temperature, air velocity and physiological monitoring are required to estimate occupational health effects and real-world implementation effectiveness.

## Figures and Tables

**Table 1:** Cooking fuel type and meal details for the three selected sites.

Description	Site A: High School Kitchen	Site B: Remand Home Kitchen	Site C: Café & Restaurant Kitchen
Stoves used	Mostly three-stone stove + some ICS	Improved cookstove (ICS) for all meals	Charcoal stove + ICS
Fuels used	Firewood	Firewood	Charcoal
Avg. number of meals prepared/day	2 (breakfast and lunch)	3 (breakfast, lunch, supper)	1 (lunch only)
Typical meal types	Tea, matooke, rice, beans	Porridge, posho, beans	Matooke, posho, meat, beans, groundnuts
Meal timing (typical start–end)	Breakfast: 07:00–10:00 Lunch: 08:00/09:00–13:00	Breakfast: 06:00–08:00 Lunch: 06:00–12:00 Supper: 08:00–15:30	Lunch: 07:00–14:00
Other kitchen activities	Food preparation	Washing, cleaning, chapati making (sometimes)	Food prep, washing
Total kitchen floor area	11.096 m^2^	40.98 m^2^	8.64 m^2^
Ceiling height	3 m	3 m	2.8 m
Approximate volume	33.3 m^3^	122.9 m^3^	24.2 m^3^
Typical cooks	2	1–2	1
Approximate cook density	0.18 cooks/m^2^	0.02–0.05 cooks/m^2^	0.12 cooks/m^2^
Material finishes	Plastered masonry walls with substantial smoke/soot deposition, corrugated metal sheet roof with exposed timber trusses, cement floor	Masonry walls with two-thirds ceramic tile finish, pitched corrugated metal sheet roofing with steel roof frames, tiled floor	Unfinished masonry block walls and floor, corrugated metal sheet roof with exposed timber trusses, red clay brick-tiled floor.
Ventilation/openings	2.16 m^2^ window + door + perforated ventilation block openings	Two 1.02 m^2^ windows + door + 3.42 m^2^ perforated ventilation block openings	1.08 m^2^ window + door + high-level ventilation openings

Note: Typical staffing is taken from the technical memo and does not represent continuously measured occupancy. Ventilation and air change rates were not measured.

**Table 2:** (**a**) Cooking duration and meals served for baseline and active e-cooking days. (**b**) Cooking duration normalised by the number of people served.

(**a**)									
**Kitchen**	**Meal**	**Baseline Mean (min)**	**Active E-Cooking Days Mean (min)**	**Time Saved (min)**	**% Reduction**	**People Served**		**Number of Meals**	
						**Baseline**	**Intervention**	**Baseline**	**Intervention**
**Site A**	Breakfast	113.73	35	78.73	69%	~50 (B,L)	~20 (B,L,S)	15	10
	Lunch	264.53	121.82	142.72	54%			15	11
	Supper	—	53	—	—			—	1
**Site B**	Breakfast	128	67.71	60.29	47%	~235 (B,L,S)	~205 (B,L,S)	15	9
	Lunch	203.33	96.43	106.9	53%			15	9
	Supper	117.33	89	28.33	24%			15	7
**Site C**	Lunch	370.8	204.83	165.97	45%	~65 (L)	~27 (L)	15	18
(**b**)									
**Site/Meal**	***n*** **(B/I)**	**Baseline min/100, Mean ± SD**		**Active min/100, Mean ± SD**			**Change (95% CI)**		***p***
A breakfast	15/10	227.6 ± 89.4		175.9 ± 85.5			−51.7 (−125.8 to 22.4)		0.161
A lunch	15/11	533.4 ± 139.3		609.5 ± 334.4			76.1 (−155.9 to 308.2)		0.490
B breakfast	15/9	54.3 ± 19.3		34.5 ± 18.3			−19.9 (−36.4 to −3.3)		0.021
B lunch	15/9	86.6 ± 51.6		52.9 ± 24.9			−33.8 (−66.4 to −1.1)		0.043
B supper	15/9	50.0 ± 30.5		44.7 ± 16.2			−5.3 (−25.1 to 14.4)		0.581
C lunch	15/18	844.5 ± 531.6		757.1 ± 373.5			−87.4 (−423.6 to 248.8)		0.597

Note: Means are based on recorded days (*n* = 15 per baseline phase; active use *n* = 15 at Site A, *n* = 9 at Site B and *n* = 18 at Site C). Site A had no baseline supper. Minutes per 100 people = (meal cooking duration ÷ people served) × 100. Positive changes indicate longer cooking time per 100 people on active use days.

**Table 3:** (**a**) Temperature summary across baseline and active e-cooking days for the three sites. (**b**) Near-stove daily mean temperature: day-level variability and uncertainty.

(**a**)									
**Site**	**Logger Location**	**Baseline Days**	**E-Cooking Days**	**Daily Mean T Baseline**	**Daily Mean T Intervention**	**Change**	**Daily Max T Baseline**	**Daily Max T Intervention**	**Change**
**Site A**	Near cookstove	17	15	31.29 °C	25.59 °C	−5.70 °C	46.97 °C	28.75 °C	−18.21 °C
**Site A**	Ambient kitchen	17	15	29.85 °C	25.58 °C	−4.27 °C	36.34 °C	28.69 °C	−7.65 °C
**Site A**	Outside kitchen	17	15	27.36 °C	24.22 °C	−3.14 °C	32.45 °C	28.66 °C	−3.79 °C
**Site B**	Near cookstove	17	9	29.69 °C	28.05 °C	−1.64 °C	34.77 °C	32.15 °C	−2.62 °C
**Site B**	Ambient kitchen	17	9	29.69 °C	27.61 °C	−2.08 °C	34.06 °C	31.83 °C	−2.23 °C
**Site B**	Outside kitchen	17	9	26.73 °C	25.50 °C	−1.23 °C	40.72 °C	39.22 °C	−1.50 °C
**Site C**	Near cookstove	17	18	30.68 °C	26.63 °C	−4.05 °C	37.72 °C	31.72 °C	−6.01 °C
**Site C**	Ambient kitchen	17	18	29.31 °C	27.31 °C	−2.00 °C	34.80 °C	31.90 °C	−2.91 °C
**Site C**	Outside kitchen	17	18	27.16 °C	25.81 °C	−1.35 °C	33.15 °C	31.79 °C	−1.36 °C
(**b**)									
**Site**	**Days (B/I)**	**Baseline SD, °C**		**Active SD, °C**		**95% CI for Phase Difference, °C**			***p***
A	17/15	1.77		1.44		−6.81 to −4.50			<0.001
B	17/9	0.88		0.96		−2.46 to −0.82			0.001
C	17/18	1.88		2.73		−5.66 to −2.44			<0.001

Day is the inferential unit. *p*-values are from two-sided unpaired Welch tests. The differences are site-specific associations and do not isolate cooking technology from temporal or operational change.

**Table 4:** Relative humidity summary across baseline and active e-cooking days for ambient kitchen environment across the three sites.

Site	Phase	Daily Mean RH	Mean Daily Min RH	Mean Daily Max RH
Site A	Baseline	54.1%	35.6%	68.4%
Site A	Intervention	64.5%	52.4%	73.7%
Site B	Baseline	62.1%	45.8%	87.6%
Site B	Intervention	61.8%	41.8%	82.9%
Site C	Baseline	56.2%	38.3%	73.2%
Site C	Intervention	59.5%	43.8%	75.4%

**Table 5:** Near-stove daily mean weather-adjusted ΔHI.

Site	Days (B/I)	Baseline, °C Mean ± SD	Active, °C Mean ± SD	Change, °C (95% CI)	*p*
A	17/15	11.63 ± 1.58	4.56 ± 1.00	−7.07 (−8.02 to −6.13)	<0.001
B	17/7	9.62 ± 1.07	8.02 ± 1.09	−1.60 (−2.68 to −0.53)	0.007
C	17/18	10.15 ± 2.09	5.62 ± 2.23	−4.52 (−6.01 to −3.04)	<0.001

ΔHI = logger Heat Index minus concurrent Kampala weather station Heat Index. Day is the inferential unit; *p*-values are from two-sided unpaired Welch tests.

**Table 6:** Weather-matched intervention period environmental sensitivity.

Site	Weather-Matched Active Use Days	All Intervention Days	Active Use Change in ΔHI, °C	All-Days Change in ΔHI, °C
A	15	20	−7.11	−6.97
B	7	20	−1.65	−1.71
C	18	20	−4.36	−4.30

The sensitivity uses continuous environmental logger data only. Fuel use, cooking activity, outages, stove stacking, workload and thermal comfort responses are unknown on non-active days; it is therefore not an intent-to-treat or implementation fidelity analysis.

## Data Availability

The dataset supporting the findings of this study is available from the corresponding author upon reasonable request.
